# PTGS2 Is Involved in Osteonecrosis of the Femoral Head and Bone Marrow Edema

**DOI:** 10.1155/ijog/8835132

**Published:** 2025-10-31

**Authors:** Chongsen Lin, Ran Li, HongDuo Lu, HuiZi Wang, HongYang Li, Hanjun Fang, Hongyu Tang, HaiQuan Liu

**Affiliations:** ^1^ Shenzhen Luohu Hospital of Traditional Chinese Medicine (Shenzhen Hospital, Shanghai University of Traditional Chinese Medicine), Shenzhen, China; ^2^ Huizhou Hospital of Guangzhou University of Chinese Medicine (Huizhou Hospital of Traditional Chinese Medicine), Huizhou, China; ^3^ Guangzhou University of Chinese Medicine, Guangzhou, Guangdong Province, China, gzucm.edu.cn; ^4^ The First Affiliated Hospital of Guangzhou University of Chinese Medicine, Guangzhou, Guangdong, China, gzhmc.edu.cn

**Keywords:** bone marrow edema, osteonecrosis of the femoral head, pain, PTGS2

## Abstract

**Background:**

Osteonecrosis of the femoral head (ONFH) is a challenging global health issue with an unclear pathogenesis, complicating the development of effective treatment strategies. Bone marrow edema (BME) is a critical imaging indicator of ONFH progression, yet its underlying mechanisms remain poorly understood.

**Methods:**

Bioinformatics was employed to identify gene characteristic of BME in ONFH patients. Expression of PTGS2 was validated in these patients through Western blot and ELISA assays. Clinical relevance was assessed by analyzing the correlation between PTGS2 expression levels and pain severity as well as the timing of total hip replacement surgery. In addition to GSE74089, we externally validated PTGS2 in an independent GEO cohort (GSE123568, human serum; GPL15207) using single‐gene receiver operating characteristic (ROC) analysis.

**Results:**

Six hundred and eighty‐eight overlapping targets were identified for ONFH and BME, with 15 key targets shared with XLGBC, including PTGS2, IGFBP3, MCL1, TNF, F7, PLA2G4A, PRKCA, MMP1, and PTGER3. GO and KEGG enrichment analyses indicated that XLGBC exerts its effects mainly through pathways related to inflammation, pain, angiogenesis, and bone metabolism, notably involving VEGF signaling, arachidonic acid metabolism, and MAPK pathways. Molecular docking revealed strong binding between XLGBC compounds and the target genes. ELISA results indicated that higher PTGS2 levels correlated with increased pain severity in ONFH patients, and Western blot analysis showed significantly elevated PTGS2 in ONFH patients compared to controls, with levels decreasing after XLGBC treatment. Patients with higher PTGS2 expression showed shorter times to hip replacement surgery, suggesting faster disease progression. In the external cohort (GSE123568), PTGS2 showed good diagnostic discrimination for ONFH versus controls (AUC = 0.86), supporting the robustness of our bioinformatics findings.

**Conclusions:**

PTGS2 is an important gene in ONFH with BME, influencing pain and disease progression. Monitoring PTGS2 expression may help to assess symptom severity and inform surgical timing in ONFH patients.

## 1. Introduction

Osteonecrosis of the femoral head (ONFH) is a challenging and refractory bone disease with an unclear pathogenesis, making its diagnosis and treatment difficult [1]. Current diagnostic approaches primarily rely on characteristic imaging changes [2], particularly magnetic resonance (MR) manifestations such as bone marrow edema (BME) [3].

BME frequently accompanies ONFH during its progression and often signals structural instability within the femoral head [4–7]. This instability increases the risk of femoral head collapse, a critical turning point in the disease’s progression and a major risk factor for further degeneration [8, 9]. The presence of BME can make it harder to accurately judge the edges of necrotic tissue, which lowers the rate of ONFH detection and delays the right treatment, making the patient’s pain worse and adding to the overall cost [10, 11]. While BME is sometimes self‐healing, its identification on MR imaging should prompt early intervention to prevent femoral head collapse, improving patient outcomes.

Traditional Chinese medicine (TCM) has unique advantages in the treatment of ONFH with certain herbal formulations showing efficacy in clinical practice. Chinese guidelines recommend Chinese medicine that activates blood circulation and removes blood stasis and tonifies the kidneys and bones as one of the pharmacological treatments for ONFH [11]. Among the nonsurgical interventions, experts unanimously recommend Xianling Bone Guarantee Capsule (XLGBC) as a TCM treatment [12]. XLGBC can increase bone density, alleviate symptoms, and determine the efficacy of the treatment, with fewer side effects, and a rich source of medication. Its main herbal functions are to nourish the liver and tonify the kidney, invigorate the blood, and regulate the collaterals [13]. The drug was officially approved by the China Food and Drug Administration as an over‐the‐counter drug for the treatment of aseptic osteonecrosis, osteoporosis, osteoarthritis, and bone fractures. Clinical practice has shown that XLGBC has good therapeutic and preventive effects in the whole process of ONFH, including during the presence of BME [14].

In this study, bioinformatics approaches were employed to identify key target proteins associated with ONFH and BME. These targets were then validated through Western blot (WB) and enzyme‐linked immunosorbent assay (ELISA) methods. Furthermore, clinical trials were conducted to explore the relationship between identified protein expression, patient‐reported pain levels, and the timing of total hip replacement surgery. This study provides a basis for further understanding the potential mechanism of the role of the BME in ONFH progression, providing a potential framework for improved diagnostic and therapeutic strategies in clinical practice.

## 2. Materials and Methods

### 2.1. Bioinformatics Section

#### 2.1.1. Screening of the Active Compounds and the Target Proteins

XLGBC is composed of six TCMs: Yinyanghuo, Bailian, Buguzhi, Shudihuang, Danshen, and Zhimu (Table S1). Active compounds and protein targets of the above six TCMs were screened in the TCMSP database (https://old.tcmsp-e.com/tcmsp.php). Additional literature was consulted to validate and supplement the identified active compounds and targets. Screening criteria for bioactive compounds were as follows: oral bioavailability (OB) ≥ 30*%* and drug‐likeness (DL) value ≥ 0.18.

#### 2.1.2. Standardization of the Gene Names for the Target Proteins

To understand the potential targets of TCM active compounds, we used the UniProt (https://www.uniprot.org/) database to convert the target protein name to the official symbol formats for gene targets. Log in to the PubChem database (https://pubchem.ncbi.nlm.nih.gov/), retrieve the canonical SMILES of active compounds in the six Chinese medicines, and predict the corresponding gene targets using smart chemical expression in the SwissTargetPrediction database (http://swisstargetprediction.ch/). The intersection of the prediction results of the two databases is the name of the gene target of XLGBC.

#### 2.1.3. The Common Target of ONFH and BME

To screen for disease targets in ONFH, we searched the Gene Expression Omnibus database (https://www.ncbi.nlm.nih.gov/geo/) using GSE74089 as a search term. After downloading the platform file GPL13497 and Series Matrix file, we performed differential analysis of the GSE74089 dataset file (limma package) and used the genes with differential expression between the obtained healthy and ONFH samples as ONFH disease targets.

To identify targets for BME, the GeneCards database (https://www.genecards.org/) was searched using relevant keywords “bone marrow edema,” “bone marrow oedema,” and “bone marrow edema,” respectively. Duplicates were removed, and the common targets between ONFH and BME were identified for further analysis.

#### 2.1.4. Intersection Between Active Compounds and Disease Targets

The intersection targets between XLGBC gene targets and disease targets for ONFH and BME were identified. These common targets represent the key therapeutic targets of XLGBC in treating ONFH associated with BME.

#### 2.1.5. Active Ingredient–Target Network

The active ingredients and coacting targets of XLGBC were imported into Cytoscape software to construct the network diagram of active ingredients and coacting targets, in which nodes represent active ingredients and targets, and straight lines represent interactions between them.

#### 2.1.6. Gene Ontology (GO) and Kyoto Encyclopedia of Genes and Genomes (KEGG) Enrichment Analyses

To understand the functions and pathways related to these gene targets, enrichment analysis on these genes was performed. The “clusterProfiler” package was used for GO functional enrichment analysis and KEGG pathway enrichment analysis [15].*p* values < 0.05 were considered significant enrichment.

#### 2.1.7. Machine Learning for Target Screening

In order to further screen the important targets, machine learning algorithms were applied. The random forest (RF) and support vector machine–recursive feature elimination (SVM‐RFE) recursive feature elimination algorithm “e1071” based on the expression matrix of the important targets in the GSE74089 dataset to screen the core targets [16, 17]. In order to obtain more stringent core targets, the core targets obtained by the two methods were selected as the final set of feature targets.

#### 2.1.8. Verifying the Correlations Between Feature Genes

GeneMANIA (https://genemania.org/) was used to explore physical and genetic interactions between the feature genes identified by machine learning. Coexpression analysis was performed using the “igraph” package to further validate the relationships between the expression levels of these genes in ONFH samples from the GSE74089 dataset.

#### 2.1.9. Molecular Docking Verification

The 2D structures of the key active compound in SDF format files were downloaded from the PubChem database. And ChemOffice software was used to build the 3D structure of the compound and save it in mol2 format and minimize its energy. The 3D structure PDB file of the target gene was downloaded from the PDB database (https://www.rcsb.org/), and PyMOL software was used to dehydrate and hydrogenate the protein and then saved as a PDB file. Molecular docking was conducted using AutoDock Vina to convert the format of active compounds and target proteins into PDBQT format, and binding energies below −5.0 kJ/mol were considered indicative of strong binding between the active compounds and the target proteins.

### 2.2. Clinical Specimen Investigation

This investigation, ethically sanctioned by the Institutional Review Board of the First Affiliated Hospital of Guangzhou University of Chinese Medicine under the protocol JY2024‐041, was conducted from May 2023 to May 2024 within the department specializing in ONFH at the aforementioned hospital. Sixty patients diagnosed with ONFH, who were hospitalized during the study period, were randomly stratified into two distinct cohorts in accordance with the predefined inclusion and exclusion criteria. The first cohort, consisting of 30 patients, was subjected to a regimen of standard care. The second cohort, also comprising 30 patients, received an escalated treatment protocol that included the administration of XLGBC in addition to the standard care. The therapeutic intervention period for both cohorts was 6 months. A control cohort, consisting of 30 individuals matched for age and sex, was concurrently established to serve as a comparator. Clinical parameters for assessment encompassed visual analog scale (VAS) scores and the determination of PTGS2 levels.

Inclusion criteria are as follows: (1) patients with a primary diagnosis of ONFH, (2) age between 18 and 65 years, (3) receipt of conservative or surgical treatment in the hospital, (4) absence of other medical conditions such as ankylosing spondylitis or rheumatoid arthritis, and (5) a history of hormonal treatment.

Exclusion criteria are as follows: (1) patients with hypertension, diabetes mellitus, or coronary artery disease; (2) history of traumatic fracture of the hip; and (3) patients unwilling to cooperate with the survey and poor compliance.

Human serum blood collection conditions are as follows: fasting, with blood drawn from the antecubital vein in the morning. Blood samples were collected in EDTA VACUETTE tubes and centrifuged at 4°C at 6000 rpm for 10 min. Plasma was immediately separated and stored in liquid nitrogen at −80°C for subsequent use.

### 2.3. WB

Extracted cartilage PTGS2 was separated by 12% SDS‐PAGE and transferred onto PVDF membranes (Millipore). The membranes were then blocked with 5% skim milk in TBST for 2 h at room temperature and further incubated with primary antibodies against alpha‐2‐HS‐glycoprotein and cytokine‐like protein 1 overnight at 4°C. After washing (5 min × 4) with TBST, the membranes were incubated with 1:5000‐diluted goat anti‐mouse (or anti‐rabbit) HRP‐conjugated secondary antibodies for 1 h at room temperature. After an additional four washes, the membranes were developed by ECL kit. WO⁃G9413B gel imaging system was used to take photos, and elpro32 software was used to extract the gray scale value of protein bands, and *β*‐actin was used as a reference to calculate the target protein expression.

### 2.4. ELISA

The content of PTGS2 in serum samples was detected using the human PTGS2 Protein ELISA Test Kit. The specific steps are as follows: prepare the serum samples from each group of patients, and dilute and configure the relevant working solutions. The diluted standards of different concentrations, serum samples from different patients, were added into a 96‐well enzyme‐linked plate in sequence and incubated at 37°C for 2 h. Next, after the liquid in the wells was aspirated, 100 *μ*L of each biotin antibody was added to each well and incubated at 37°C for 1 h. After washing three times using 200 *μ*L/well of wash buffer, 100 *μ*L of HRP‐avidin reagent was added to each well and incubated for 1 h. After washing again five times using 200 *μ*L/well of wash buffer, 90 *μ*L/well of TMB substrate was added and incubated for 30 min at 37°C, protected from light. Finally, 50 *μ*L/well of stop solution was added to each well to terminate the reaction. Detect the absorbance values of the samples and standards at 450 nm (OD450) and 540 nm (OD540), and use OD450‐OD540 to represent the absorbance of each well, and plot a standard curve with the absorbance values of the standards to calculate the concentration of PTGS2 protein in the samples.

### 2.5. Cell Culture

The murine preosteoblastic cell line MC3T3‐E1 was kindly provided by Dr. Haoran Zhu from Zhongshan Hospital of Traditional Chinese Medicine [18]. Cells were cultured in *α*‐minimum essential medium (*α*‐MEM) supplemented with 10% fetal bovine serum (FBS), 1% penicillin–streptomycin, and 1% L‐glutamine. All cells were maintained in a humidified incubator at 37°C with 5% CO₂. The cell line and culture conditions were established based on previously published methods.

### 2.6. Cell Viability Assay (CCK‐8)

To assess cytotoxicity, MC3T3‐E1 was seeded into 96‐well plates (5 × 10^3^ cells/well) and treated with various concentrations (1, 5, 10, and 50 *μ*M) of PTGS2 agonist for 24 h. After treatment, 10 *μ*L of CCK‐8 solution was added to each well and incubated for 2 h. Absorbance was measured at 450 nm using a microplate reader. All experiments were conducted in triplicate.

### 2.7. Osteogenic Induction and Alkaline Phosphatase (ALP) Staining

For osteogenic induction, MC3T3‐E1 was cultured in osteogenic differentiation medium (ODM: *α*‐MEM supplemented with 10% FBS, 50 *μ*g/mL ascorbic acid, 10 mM *β*‐glycerophosphate, and 100 nM dexamethasone) with or without 10% XLGBC‐containing serum. After 3.5 days, cells were fixed with 4% paraformaldehyde and stained using an ALP staining kit according to the manufacturer’s protocol. ALP activity was visualized and imaged under a microscope.

### 2.8. Quantitative PCR (qPCR)

Total RNA was extracted from MC3T3‐E1 using TRIzol reagent and reverse‐transcribed into cDNA using a commercial kit. qPCR was performed using SYBR Green Master Mix on a real‐time PCR system. Primer sequences for PTGS2 (forward: GCAAATTGCTGGCAGGGTTG; reverse: GCTCTGGTCAATGGAAGCCT) and Runx2 (forward: CGCCTCACAAACAACCACAG; reverse: ACTGCTTGCAGCCTTAAATGAC) were designed based on published sequences. GAPDH (forward: CTCCTCCGGGTGATGCTTTT; reverse: GAGGGATCTCGCTCCTGGAA) was used as an internal control.

### 2.9. Determination of Symptomatic Severity

Nontraumatic ONFH symptom severity was assessed using the VAS, which asks patients to rate their pain on a scale from 0 (*no pain*) to 10 (*extreme pain*).

### 2.10. Independent Datasets and External Validation of PTGS2

To validate reproducibility, the GEO dataset GSE123568 (30 ONFH, 10 controls) was analyzed. Expression values were log2‐transformed and summarized by median when multiple probes matched a gene. ROC analysis was performed to evaluate the ability of PTGS2 to discriminate ONFH from controls. The optimal cutoff was determined by Youden’s J statistic. AUCs with 95% CIs were computed (DeLong), and the Youden index defined optimal thresholds.

### 2.11. Statistical Analysis

Statistical analysis of the bioinformatics section was performed using R (V. 4.2.0) and its associated packages, whereas GraphPad Prism software (Version 7.0) was employed for the remaining statistical analyses. Student’s *t*‐test was used to assess the comparative differences between two groups, whereas one‐way analysis of variance (ANOVA) was used to ascertain significant distinctions in multiple comparisons involving three or more groups. By conventional criteria, a *p* value lower than 0.05 was deemed statistically significant. The Kaplan–Meier (K‐M) survival analysis was performed with time to total hip arthroplasty as the endpoint. *p* < 0.05 was considered a statistically significant difference.

## 3. Results

### 3.1. Identification of Active Compounds and Potential Targets in XLGBC

A total of 30 active compounds were identified from the six herbal components of XLGBC using TCMSP and literature mining. These were distributed as follows: Yinyanghuo (10), Bailian (3), Buguzhi (2), Shudihuang (1), Danshen (13), and Zhimu (7). The corresponding gene targets were 104 after removal of duplicates (Table S2). From the GEO and GeneCards databases, 2862 targets related to ONFH and 4971 related to BME were retrieved. The intersection yielded 688 shared targets relevant to ONFH with BME (Figure 1a and Table S3). Fifteen intersecting genes were identified between XLGBC targets and ONFH‐BME shared targets: PTGER3, MET, F7, PTGES, HRH1, NR1I3, TNF, PLA2G4A, PTGS2, IGFBP3, VEGFA, ACHE, MCL1, PRKCA, and MMP1 (Figure 1b). Cytoscape was used to construct a network showing interactions between 13 active compounds and the 15 core targets, revealing multicomponent and multitarget pharmacological features of XLGBC (Figure 1c).

Figure 1Identification of disease‐related and XLGBC‐related targets. (a) Venn diagram showing overlapping gene targets between ONFH and BME. (b) Venn diagram of intersecting targets between XLGBC and ONFH‐BME shared genes. (c) Active ingredient–target interaction network constructed with Cytoscape, illustrating multitarget pharmacological relationships.

**Figure:**
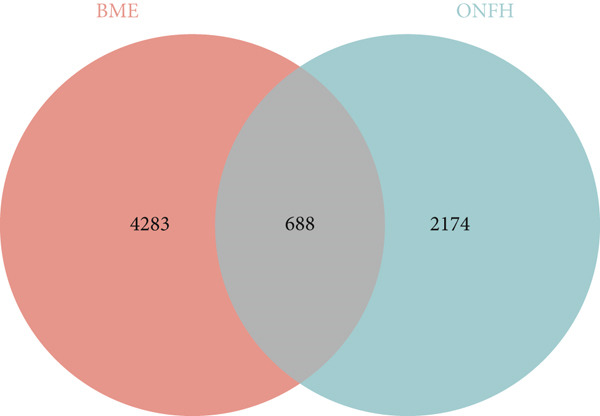
(a)

**Figure:**
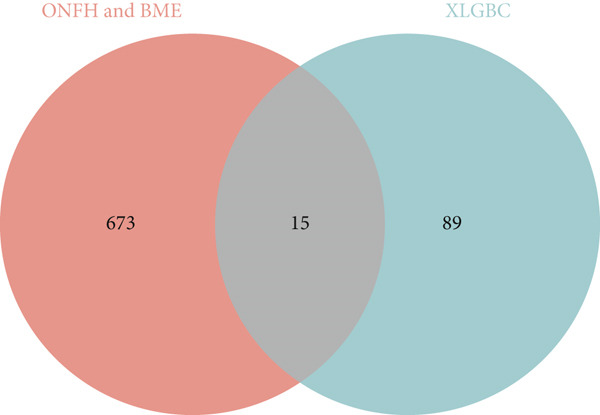
(b)

**Figure:**
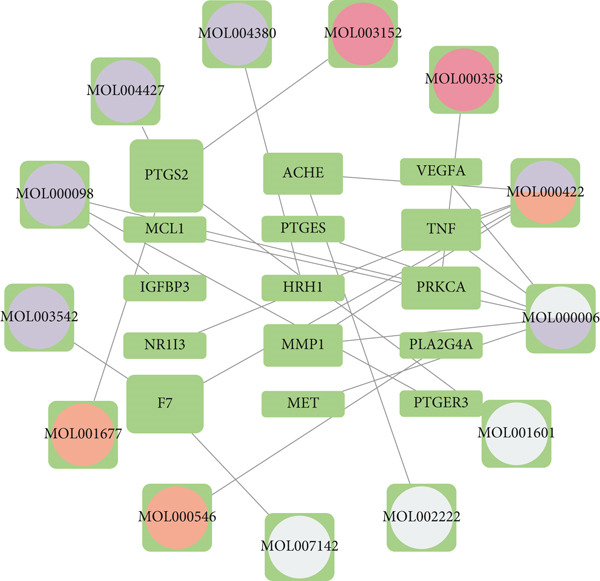
(c)

### 3.2. External Validation of PTGS2 in an Independent Cohort

In the independent serum cohort GSE123568 (30 ONFH, 10 controls), PTGS2 yielded an AUC of 0.86 (95% CI 0.74–0.97; DeLong). The Youden optimal cutoff was 8.23, giving sensitivity 0.80 and specificity 0.90. These findings independently corroborate the discovery set and support the reproducibility of PTGS2 as a diagnostic‐grade biomarker candidate (Figure 2a).

Figure 2(a) ROC curve of PTGS2 in the independent cohort GSE123568, showing good discrimination between ONFH and controls (AUC = 0.86, 95% CI 0.74–0.97; DeLong). (b) GO enrichment analysis of significant molecular function terms. (c) KEGG pathway enrichment of therapeutic targets. (d) SVM‐RFE analysis identifying 15 optimal gene features. (e) Random forest model analysis showing lowest prediction error at 15 trees. (f) Top 9 characteristic targets selected by combined ML algorithms.

**Figure:**
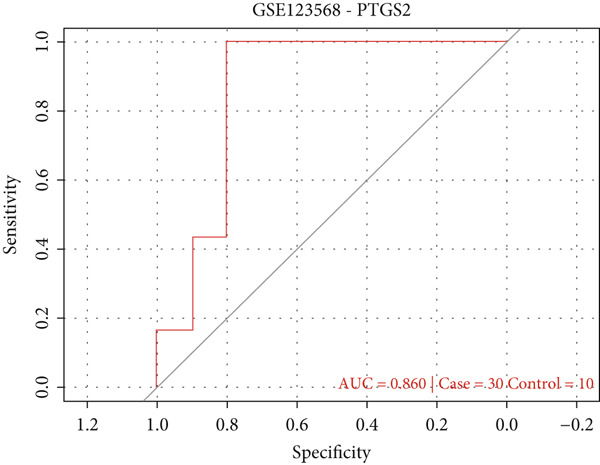
(a)

**Figure:**
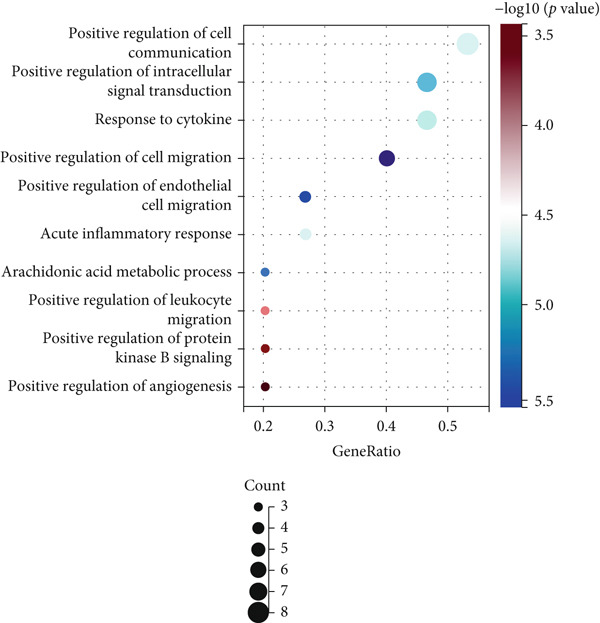
(b)

**Figure:**
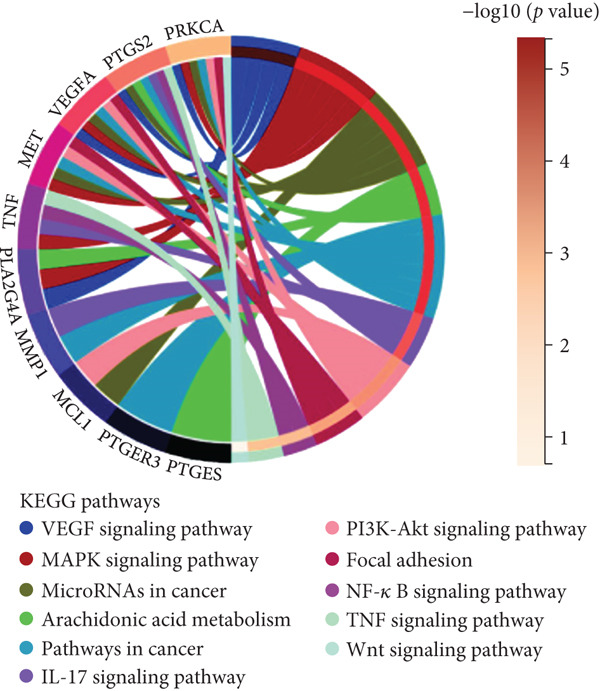
(c)

**Figure:**
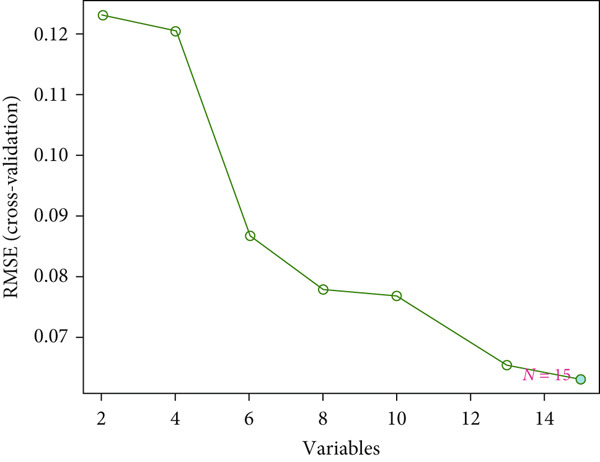
(d)

**Figure:**
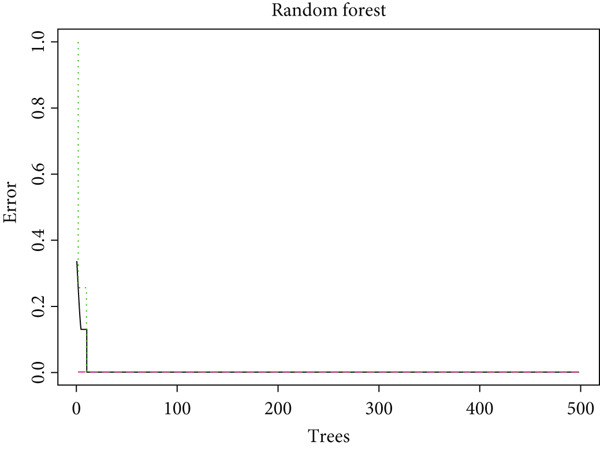
(e)

**Figure:**
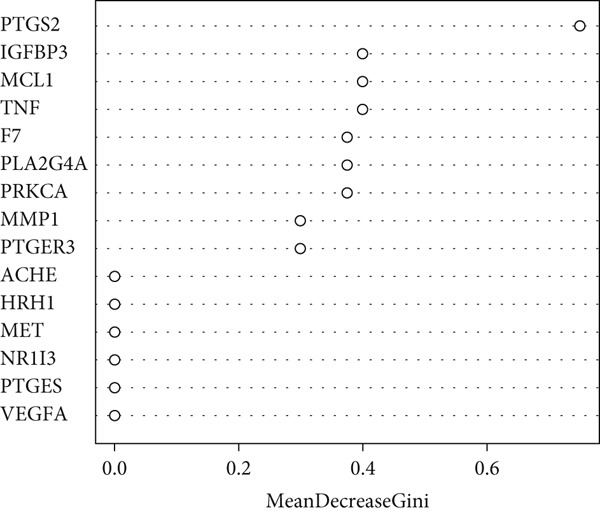
(f)

### 3.3. Functional Enrichment and Machine Learning–Based Target Prioritization

GO enrichment analysis revealed that the intersecting targets were significantly involved in several biological processes, especially those related to inflammation and cellular signaling. The top enriched GO terms included positive regulation of cellular communication, positive regulation of intracellular signaling pathways, response to cytokines, regulation of cell migration, and acute inflammatory response (Figure 2b). These biological functions are closely associated with the pathological progression of ONFH with BME. KEGG pathway analysis further revealed that the most enriched signaling pathways included the VEGF signaling pathway, the arachidonic acid metabolic pathway, and the MAPK signaling pathway (Figure 2c). These pathways play pivotal roles in angiogenesis, inflammation, and cellular stress responses, suggesting their relevance to the therapeutic mechanism of XLGBC.

To further refine the most critical targets, two machine learning models were employed: RF and SVM‐RFE. RF model performance was evaluated based on decreasing root mean square error (RMSE), and the optimal model included 15 variables. SVM‐RFE applied sequential feature ranking to identify important predictors. The intersection of these two models resulted in the selection of nine highly representative hub genes: PTGS2, IGFBP3, MCL1, TNF, F7, PLA2G4A, PRKCA, MMP1, and PTGER3 (Figures 2d, 2e, and 2f). These genes are intricately linked to inflammatory regulation, oxidative stress, vascular responses, and tissue remodeling, emphasizing their central roles in ONFH pathogenesis and the potential action targets of XLGBC.

### 3.4. PTGS2‐Centric Network and Molecular Docking Validation

GeneMANIA analysis revealed that PTGS2 is intricately linked with other machine learning–identified targets, including IGFBP3, MCL1, TNF, F7, PLA2G4A, PRKCA, and VEGFA, forming a robust gene interaction network (Figure 3a). These interactions suggest that PTGS2 may play a central regulatory role in pathways associated with inflammation, cell survival, and vascular remodeling, all of which are critical in ONFH pathophysiology.

Figure 3PTGS2‐centered functional network and compound binding verification. (a) GeneMANIA‐based PPI network illustrating key interactions of PTGS2. (b–d) Molecular docking results showing binding of XLGBC compounds (1,2,5,6‐tetrahydrotanshinone, gentisin, and asperglaucide) with PTGS2.

**Figure:**
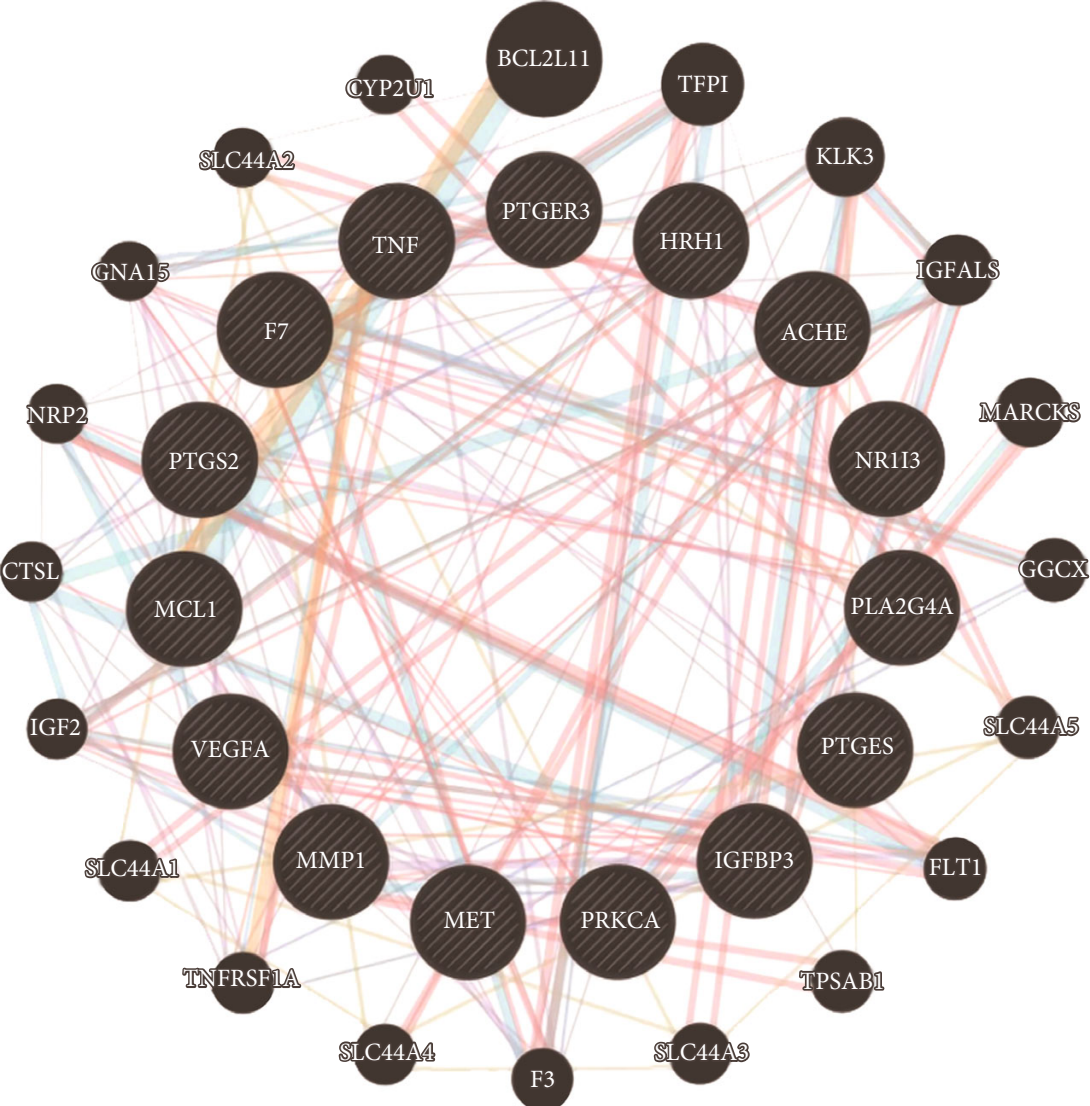
(a)

**Figure:**
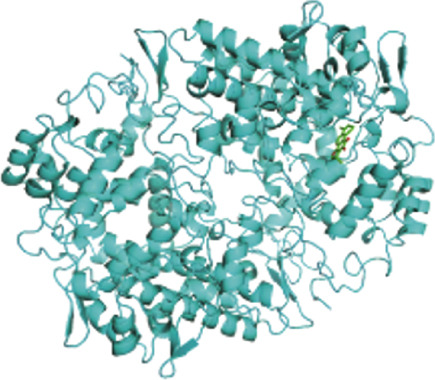
(b)

**Figure:**
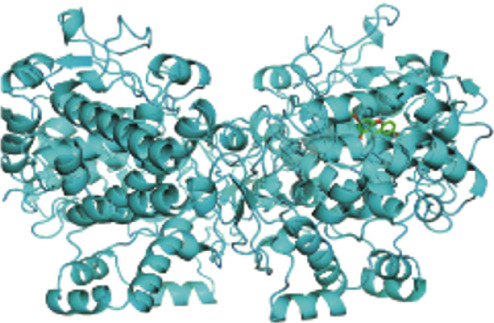
(c)

**Figure:**
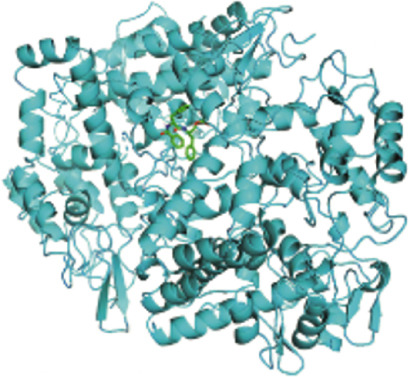
(d)

To further explore the mechanistic basis of PTGS2 regulation by XLGBC, molecular docking studies were performed between the PTGS2 protein and the top‐ranked active ingredients of XLGBC. Several compounds demonstrated strong binding affinities with PTGS2, all exhibiting binding energies less than −5.0 kJ/mol, indicating stable and favorable interactions (Figures 3b, 3c, and 3d). This result supports the hypothesis that XLGBC may exert its therapeutic effects, at least in part, through direct modulation of PTGS2 activity by its bioactive constituents.

### 3.5. Clinical Validation of PTGS2 Expression and Prognostic Relevance

WB results showed significantly higher PTGS2 protein levels in ONFH patients compared to healthy controls (*p* < 0.05), and levels significantly decreased following XLGBC treatment (*p* < 0.001) (Figure 4a,b,b). ELISA results demonstrated that PTGS2 levels were positively correlated with VAS pain scores and MDA levels, suggesting a link between PTGS2, pain severity, and oxidative stress (Figure 4c). A strong correlation was observed between PTGS2 levels and VAS scores (*r* = 0.9576, *p* < 0.0001) (Figure 4d). K‐M analysis showed that the XLGBC group had a significantly higher hip survival rate compared to controls (85.4%, 95% CI: 1.251–2.022), indicating a protective effect (Figure 4e).

Figure 4(a, b) Western blot analysis of PTGS2 protein expression in clinical serum samples. (a) Representative blot showing PTGS2 levels in normal control, ONFH patients before XLGBC treatment (pre‐XLGBC), and after treatment (post‐XLGBC). (b) Densitometric quantification of PTGS2 bands normalized to *β*‐actin.  ^∗^ *p* < 0.05;  ^∗∗∗^ *p* < 0.001. (c) ELISA analysis showing that serum PTGS2 levels increased in parallel with pain severity. (d) Positive correlation between PTGS2 and VAS score (*r* = 0.9576, *p* < 0.0001). (e) Kaplan–Meier curve showing improved hip survival in the XLGBC‐treated patient group.

**Figure:**
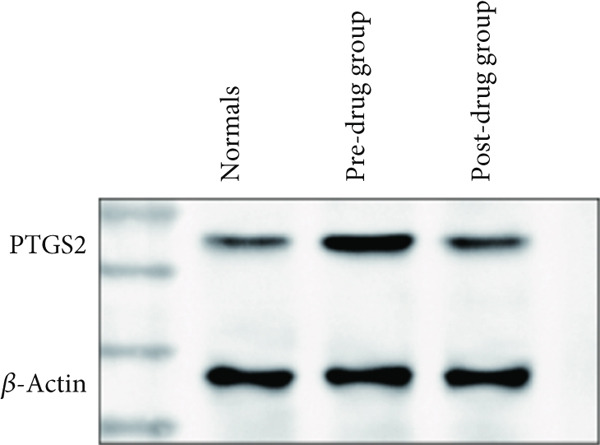
(a)

**Figure:**
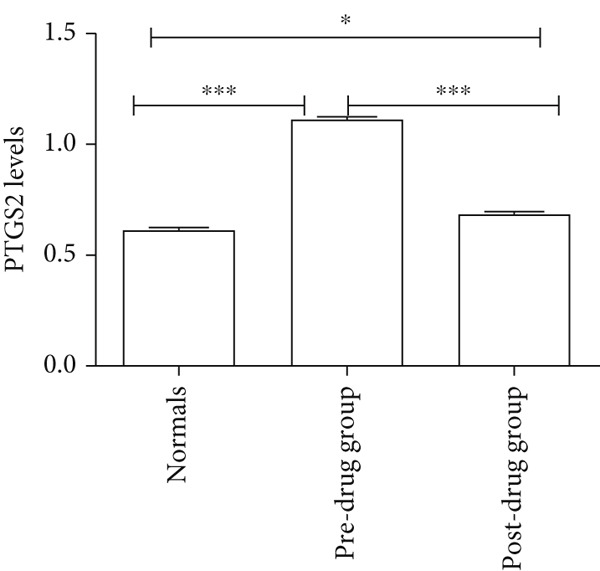
(b)

**Figure:**
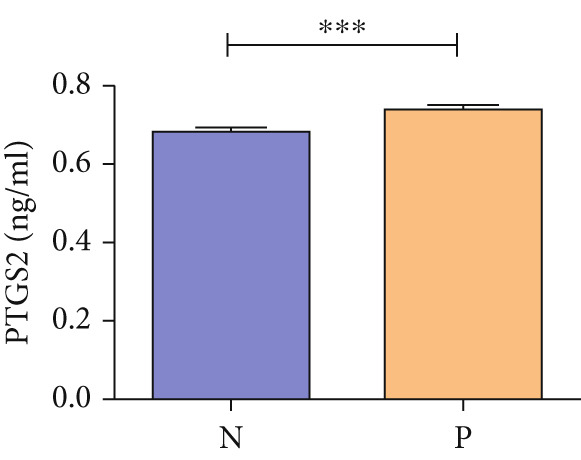
(c)

**Figure:**
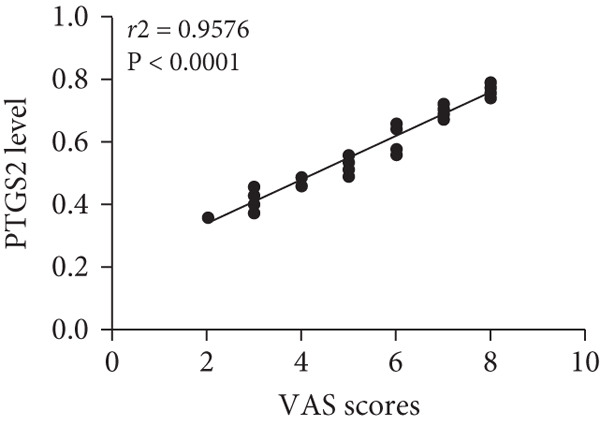
(d)

**Figure:**
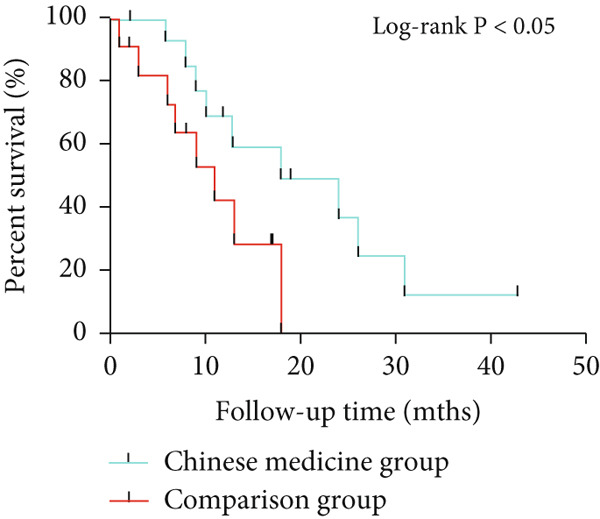
(e)

### 3.6. XLGBC Promotes Osteogenic Differentiation of MC3T3‐E1 via PTGS2 Inhibition

CCK‐8 assay confirmed that PTGS2 agonist concentrations up to 50 *μ*M had no cytotoxic effects on MC3T3‐E1 (Figure 5a). ALP staining demonstrated increased osteogenic differentiation in the XLGBC group, which was attenuated by PTGS2 activation (Figure 5b). qPCR revealed that XLGBC reduced PTGS2 mRNA levels and upregulated Runx2 expression, both of which were reversed by PTGS2 agonist treatment (Figure 5c,d,d). These findings suggest that XLGBC promotes osteogenesis through inhibition of PTGS2 signaling.

Figure 5XLGBC enhances BMSC osteogenic differentiation via PTGS2 inhibition. (a) CCK‐8 assay showing no cytotoxicity of PTGS2 agonist at selected concentrations. (b) ALP staining showing enhanced osteogenesis with XLGBC, reversed by PTGS2 activation. (c) qPCR showing decreased PTGS2 mRNA expression after XLGBC treatment, restored by PTGS2 agonist. (d) Runx2 expression increased by XLGBC and reduced by PTGS2 activation.  ^∗^ *p* < 0.05;  ^∗∗^ *p* < 0.01;  ^∗∗∗^ *p* < 0.001.

**Figure:**
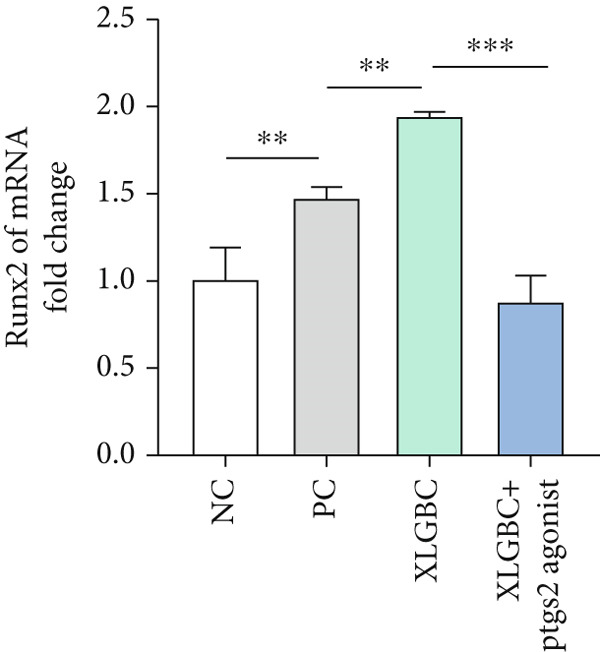
(a)

**Figure:**
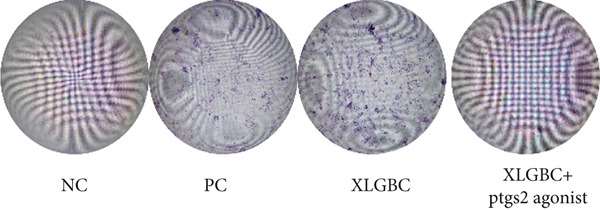
(b)

**Figure:**
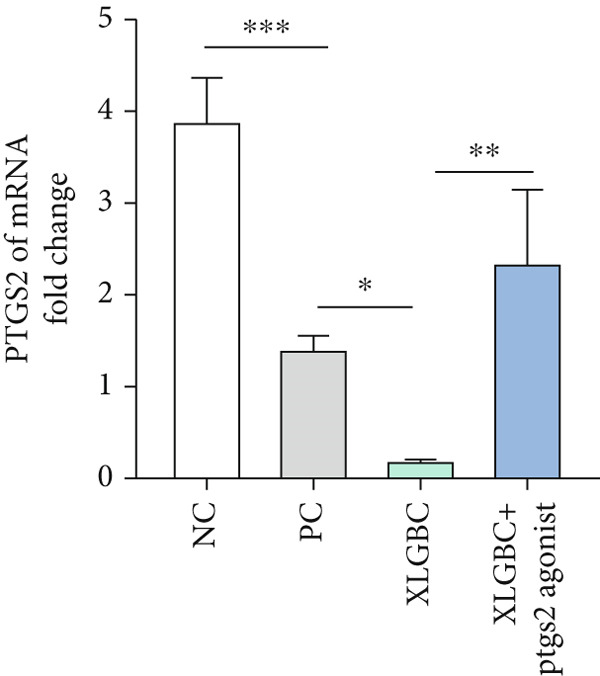
(c)

**Figure:**
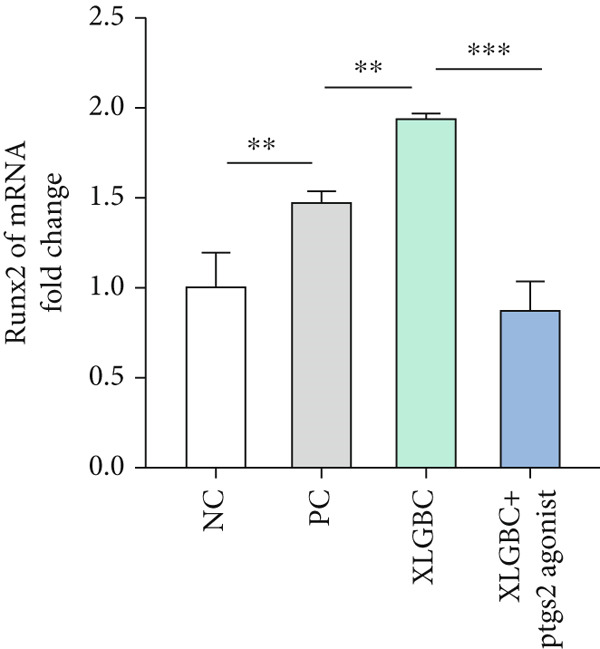
(d)

## 4. Discussion

At present, the mechanism of femoral head necrosis is not completely clear. A large number of studies have shown that BME is closely related to femoral head collapse and pain aggravation in the pathological process of ONFH [6, 19]. In this study, we aimed to explore the molecular targets associated with ONFH and BME, focusing on identifying potential therapeutic pathways to mitigate femoral head collapse. In addition to discovery in GSE74089, we externally validated PTGS2 in the independent cohort GSE123568, where it achieved strong discriminative performance (AUC = 0.86). This independent validation strengthens the reproducibility and generalizability of our findings.

To explore the molecular mechanisms underlying the therapeutic effects of XLGBC in ONFH with BME, we identified a set of common targets shared by the disease and the drug and further prioritized key genes through enrichment analysis and machine learning. Our functional analysis highlighted several pivotal pathways—VEGF signaling, arachidonic acid metabolism, and the MAPK pathway—all of which are strongly implicated in ONFH pathophysiology. Among them, VEGF signaling plays a dual role in angiogenesis and endochondral bone formation, with VEGFA acting as a critical factor in vascular remodeling and cartilage ossification, both essential to femoral head integrity [20, 21]. Disruption of this pathway has been linked to impaired bone repair and collapse.

The arachidonic acid pathway, involving PTGS2, PTGES, and PLA2G4A, is central to the synthesis of proinflammatory prostaglandins and pain mediators [22]. Meanwhile, the MAPK signaling pathway is known to regulate osteoblast differentiation, apoptosis, and stress responses, particularly in the context of cartilage injury [23].

Notably, both bioinformatics and experimental evidence converged on PTGS2 as a key effector gene. Mechanistically, PTGS2 catalyzes prostaglandin synthesis, which can sensitize nociceptors and promote pain. In parallel, prostaglandin‐mediated inflammatory signaling may synergize with VEGF‐driven vascular permeability, leading to interstitial fluid accumulation and BME. This dual role provides a plausible explanation for the observed correlation between PTGS2, pain severity, and ONFH progression. Its upregulation in ONFH may contribute to local inflammation and osteogenic suppression, thereby exacerbating pain and structural degeneration. These findings suggest that XLGBC may exert its therapeutic effect in part by downregulating PTGS2 and restoring osteogenic balance, offering a potential mechanistic explanation for its clinical benefits in ONFH with BME.

In the independent serum cohort GSE123568 (30 ONFH and 10 controls), PTGS2 distinguished cases from controls with an AUC of 0.861 (95% CI 0.763–0.938; DeLong). Using the Youden optimal threshold of 8.23, sensitivity and specificity were 0.80 and 0.90, respectively (Figure 2a); performance estimates were optimism‐corrected by 1000‐bootstrap resampling, supporting reproducibility beyond GSE74089.

PTGS2 can cause pain through multiple pathways, the most important of which is the production of prostaglandins through the arachidonic acid pathway and the production of metabolites that cause pain. PG enhances or prolongs the pain‐inducing effect of histamine, 5‐hydroxytryptamine, and other pain‐inducing substances on sensory nerve endings and enhances the sensitivity of the body to pain [24, 25]. Therefore, we hypothesized that the pain symptoms in the early stage of femoral head collapse may be closely related to PTGS2, and the degree of pain is positively correlated with the grade of BME.

Moreover, our study also involved the VEGF signaling pathway, and PTGS2 was also involved [26]. VEGFA is an active growth factor in angiogenesis, angiogenesis, and endothelial cell growth, which can induce the increase of vascular permeability. VEGFA increases vascular permeability and induces vasodilation, resulting in increased tissue blood flow [27]. Vascular permeability is essential for normal tissue homeostasis, and its imbalance can lead to tissue edema. The enhanced vascular permeability induced by VEGFA is the basis for its role in inflammation and other pathological settings. Inflammatory cells infiltrate the perilesional tissue of the femoral head, which induces microvascular hyperemia and edema and increases vascular permeability. The subsequent accumulation of interstitial fluid in the bone marrow space eventually leads to bone marrow edema [28–31]. Therefore, we believe that VEGFA plays a role in promoting inflammation and increasing vascular permeability during femoral head necrosis and BME. The presence of PTGS2 enhances this process. This makes us pay more attention to PTGS2.

Therefore, we hypothesized that PTGS2 may be a key factor in the development and progression of ONFH disease. Our experimental findings support these hypotheses. ELISA results demonstrated a positive correlation between PTGS2 expression and pain severity in ONFH patients, and increased oxidative stress markers indicated a possible interaction with the NF‐*κ*B pathway. WB analysis further validated the therapeutic effect of XLGBC in reducing PTGS2 levels, highlighting PTGS2’s potential as a biomarker and therapeutic target in ONFH complicated by BME. Clinically, these findings suggest that PTGS2 could serve not only as a biomarker reflecting inflammatory activity but also as an adjunct to MRI‐based BME evaluation for predicting collapse risk and guiding individualized treatment.

Next, we conducted clinical observations to confirm this hypothesis again. Clinically, patients with elevated PTGS2 expression exhibited higher VAS pain scores and required earlier total hip replacement, indicating faster femoral head collapse. This suggests that PTGS2 expression levels could serve as a predictor of clinical outcomes, guiding treatment timing in patients with pericollapse ONFH.

Further literature retrieval has revealed that PTGS2 is a protein secreted by osteoblasts, which acts on PGE4 and participates in pain modulation. Additionally, reduced expression of PTGS2 can enhance osteogenic differentiation capacity [32]. In our study, XLGBC can effectively inhibit the secretion of PTGS2 and promote osteogenic differentiation of BMSCs, which may constitute a potential mechanism by which it ameliorates local osteogenic insufficiency in the femoral head, thereby exerting a therapeutic effect on ONFH [33].

This study presents PTGS2 as a promising biomarker and therapeutic target in ONFH complicated by BME, yet several limitations must be acknowledged. First, the limited sample size prevented stratified analyses across different ARCO stages, restricting insights into stage‐specific relevance. We are currently collecting peripheral blood samples from patients at various ARCO stages to address this gap. Second, the cross‐sectional design cannot capture dynamic changes in PTGS2 levels; longitudinal follow‐up is required to clarify their relationship with pain progression, BME severity, and femoral head collapse. Third, while our clinical data suggest a role for PTGS2, its mechanistic involvement in VEGF and NF‐*κ*B pathways requires further confirmation through in vitro and in vivo studies. Finally, discovery relied on a single dataset (GSE74089) and BME‐associated genes from the GeneCards database, which may introduce selection bias given its literature‐based rather than experimentally curated nature. Although we mitigated these issues by applying strict inclusion criteria and validating PTGS2 in an independent cohort (GSE123568), additional independent datasets and experimental models are needed to confirm and extend these findings.

## 5. Conclusion

In summary, via network pharmacology, we identified that XLGBC may exert therapeutic effects by regulating the expression of multiple targets. Our findings further suggest that inflammation, pain, and increased vascular permeability are likely involved in the early stages of femoral head necrosis and collapse, with PTGS2 representing a key molecular mechanism. Results from ELISA and WB experiments further support the functional role of PTGS2, demonstrating that elevated PTGS2 expression correlates with higher pain levels (as indicated by VAS scores) and a shorter interval from the pericollapse stage to total hip replacement surgery. These findings indicate that PTGS2 may function as an objective biomarker for monitoring symptom severity and prognosis in ONFH patients. By elucidating the associations between BME, pain, and femoral head collapse, this study provides novel insights into the pathophysiology of ONFH and lays a foundation for future research focused on targeted therapies and optimized clinical management.

**Nomenclature**
ONFH: osteonecrosis of the femoral head
BME: bone marrow edema
XLGBC: Xianling Bone Guarantee Capsule
VEGF: vascular endothelial growth factor
MAPK: mitogen‐activated protein kinase
MR: magnetic resonance
TCM: traditional Chinese medicine
WB: Western blot
ELISA: enzyme‐linked immunosorbent assay
GO: Gene Ontology
KEGG: Kyoto Encyclopedia of Genes and Genomes
RF: random forest
SVM‐RFE: support vector machine–recursive feature elimination

## Ethics Statement

The study protocol, designated as JY2024‐041, was subjected to rigorous review and obtained approval from the Institutional Review Board of the First Affiliated Hospital of Guangzhou University of Chinese Medicine, adhering to the ethical standards for medical research involving human subjects as delineated in the 1964 Declaration of Helsinki and its subsequent amendments. Written informed consent was secured from all participants prior to their involvement in the study, ensuring their voluntary participation and comprehension of the research procedures and potential implications.

## Consent

All authors agree to publish this paper.

## Disclosure

All authors reviewed and commented on earlier versions of the manuscript and approved the final version for submission.

## Conflicts of Interest

The authors declare no conflicts of interest.

## Author Contributions

Chongsen Lin and Ran Li contributed to the original draft writing, validation, formal analysis, visualization, software development, methodology design, conceptualization, and data curation. HaiQuan Liu, Hanjun Fang, and Hongyu Tang were responsible for methodology support, manuscript review and editing, funding acquisition, resource provision, supervision, and project administration. HongDuo Lu contributed to resources, data curation, investigation, formal analysis, validation, and software. HuiZi Wang and HongYang Li were involved in resources, data curation, formal analysis, and software. Chongsen Lin and Ran Li contributed equally to this work and share first authorship.

## Funding

The study was funded by the National Research Special Project for the Inheritance and Innovation of Traditional Chinese Medicine (2022QN07 and 2023QN17), Guangdong Provincial Administration of Traditional Chinese M (20231122), and Talent‐Bidding Project for Key Tasks of the Third Clinical Medical College of Guangzhou University of Chinese Medicine.

## Supporting information


**Supporting Information** Additional supporting information can be found online in the Supporting Information section. Table S1: The components of traditional Chinese medicines in Xianling Gubao Capsule. Table S2: The active compounds and targets of Xianling Gubao Capsule. Table S3: The common targets of ONFH and BME.

## Data Availability

The data generated in this study are available from the corresponding authors upon request.
