# Supplementary material for: PTGS2 Is Involved in Osteonecrosis of the Femoral Head and Bone Marrow Edema
Source: Int J Genomics. 2025 Oct 31;2025:8835132. doi: 10.1155/ijog/8835132 (PMC12577566; doi:10.1155/ijog/8835132)
Supplement: Supplementary file 1 — Supporting Information Additional supporting information can be found online in the Supporting Information section. Table S1: The components of traditional Chinese medicines in Xianling Gubao Capsule. Table S2: The active compounds and targets of Xianling Gubao Capsule. Table S3: The common targets of ONFH and BME. [file IJOG-2025-8835132-s001.zip › Supplementary Table1.docx]

**Table 1** The components of traditional Chinese medicines in Xianling Gubao Capsule

| NO. | Chinese name | Latin name |
| --- | --- | --- |
| 1  2  3  4  5  6 | Yin-Yang-Huo  Xu-Duan  Bu-Gu-Zhi  Di-Huang  Dan-Shen  Zhi-Mu | Epimrdii Herba  Dipsaci Radix  Fructus Psoraleae  Rehmanniae Radix Praeparata  Radix Salviae  Anemarrhenae Rhizoma |
