# Supplementary material for: PTGS2 Is Involved in Osteonecrosis of the Femoral Head and Bone Marrow Edema
Source: Int J Genomics. 2025 Oct 31;2025:8835132. doi: 10.1155/ijog/8835132 (PMC12577566; doi:10.1155/ijog/8835132)
Supplement: Supplementary file 1 — Supporting Information Additional supporting information can be found online in the Supporting Information section. Table S1: The components of traditional Chinese medicines in Xianling Gubao Capsule. Table S2: The active compounds and targets of Xianling Gubao Capsule. Table S3: The common targets of ONFH and BME. [file IJOG-2025-8835132-s001.zip › Supplementary Table2.docx]

**Table 2:** The active compounds and targets of Xianling Gubao Capsule

| Chinese herb | active compound | target |
| --- | --- | --- |
| Yinyanghuo | 8-Isopentenyl-kaempferol | F7,KCNH2,SCN5A,GABRA1 |
|  | Anhydroicaritin | ESR2 |
|  | C-Homoerythrinan, 1,6-didehydro-3,15,16-trimethoxy-, (3.beta.) | SLC6A2,CHRM5,ADRA2B,OPRD1,ADRA2A,HTR3ACHRM1,HRH1,ADRA1A,CHRM2,OPRM1,CHRM4,ADRB1,ADRA1B,CHRM3,ADRA1D,SLC6A3 |
|  | Chryseriol | NOS2,CHEK1,MAPK14,AR,GSK3B |
|  | DFV | SLC6A4,RXRA,ESR1,PTGS1,ADRB2 |
|  | Icariside A7 | PTGS2 |
|  | kaempferol | CYP1A2,IKBKB,BCL2,MAPK8,CYP1A1,SELE,AHR,NR1I3,AKR1C3,ACHE,CYP3A4,CYP1B1 |
|  | luteolin | MMP2,XDH,HMOX1,MET,MAPK1,MDM2,EGFR,IL2,ERBB2,TOP2A,BCL2L1,PTGES,PPARG,RELA,TYR,TP53,TNF,INSR,CASP9,AKT1,CASP7,VEGFA,MCL1,MMP1,CASP3,MMP9,TOP1 |
|  | quercetin | PPARD,PTGER3,PLAU,MMP3,PPARA,PRKCB,CASP8,CHEK2,HIF1A,NOS3,SERPINE1,RAF1,MPO,CHUK,IGFBP3,F3,CTSD,PRKCA |
|  | sitosterol | NR3C2，PGR |
| Xuduan | beta-sitosterol | CHRM4,SLC6A4,CASP8,PRKCA,CHRM2,CASP3 |
|  | Gentisin | MAPK14,NOS2,PTGS2,CHEK1,ESR2 |
|  | sitosterol | NR3C2,PGR |
| Buguzhi | Isobavachin | PTGS1,NOS2,ESR2,ESR1 |
|  | Stigmasterol | ADRA2A,RXRA |
| Danshen | 1,2,5,6-tetrahydrotanshinone | SLC6A4,CHRM5,RXRA,OPRD1,HTR3A,CHRM1,ADRA1A,CHRM2,OPRM1,CHRM4,ADRA1B,PTGS2,CHRM3,PTGS1,ADRA1D,SCN5A,GABRA1,SLC6A3 |
|  | 2-(4-hydroxy-3-methoxyphenyl)-5-(3-hydroxypropyl)-7-methoxy-3-benzofurancarboxaldehyde | NOS2,MAPK14,ESR2,GSK3B |
|  | 4-methylenemiltirone | ADRA2C,ADRA2A |
|  | Danshenol A | KCNH2 |
|  | dan-shexinkum d | CHEK1 |
|  | Dehydrotanshinone II A | ESR1,AR |
|  | luteolin | MMP2,XDH,HMOX1,MET,MAPK1,MDM2,EGFR,IL2,ERBB2,TOP2A,ICAM1,BCL2L1,PTGES,PPARG,RELA,GSTP1,TYR,TNF,INSR,CASP9,PCNA,AKT1,NFKBIA,CASP7,MCL1,MMP1,CASP3,MMP9,TOP1 |
|  | Poriferasterol | NR3C2,PGR |
|  | przewalskin a | NR3C1 |
|  | salvianolic acid j | F7 |
|  | salviolone | SLC6A2,ADRA2B |
|  | sugiol | ACHE |
|  | tanshinone iia | CYP1A2,BCL2,EDNRA,ECE1,CYP3A4 |
| Shudihuang | Stigmasterol | SLC6A2,CHRM1,CHRM3,CHRM2 |
| Zhimu | Anemarsaponin F_qt | NR3C1 |
|  | Anhydroicaritin | KDR,CHRM5,CHEK1,MAPK14,CHRM3,ESR1,ADRB2,ESR2,GSK3B |
|  | asperglaucide | KCNH2,PTGS2 |
|  | diosgenin | FASN,HIF1A,TP53,PLA2G4A |
|  | Hippeastrine | OPRD1,OPRM1 |
|  | kaempferol | SLC6A2,XDH,NOS2,CYP1A2,IKBKB,BCL2,F7,MAPK8,CYP1A1,CHRM1,PPARG,RELA,AHR,PGR,CHRM2,NR1I3,TNF,INSR,AKT1,AKR1C3,PTGS1,NR1I2,AR,ACHE,MMP1,CYP3A4,CYP1B1 |
|  | Stigmasterol | NR3C2,PLAU,ADRA2A,ADRB1,SLC6A3 |
