# Supplementary material for: PTGS2 Is Involved in Osteonecrosis of the Femoral Head and Bone Marrow Edema
Source: Int J Genomics. 2025 Oct 31;2025:8835132. doi: 10.1155/ijog/8835132 (PMC12577566; doi:10.1155/ijog/8835132)
Supplement: Supplementary file 1 — Supporting Information Additional supporting information can be found online in the Supporting Information section. Table S1: The components of traditional Chinese medicines in Xianling Gubao Capsule. Table S2: The active compounds and targets of Xianling Gubao Capsule. Table S3: The common targets of ONFH and BME. [file IJOG-2025-8835132-s001.zip › Supplementary Table3.docx]

**Table 3:** The common targets of ONFH and BME

| Target names | Description | logFC |
| --- | --- | --- |
| CD44 | CD44 Molecule (Indian Blood Group) | 1.658059 |
| MEFV | MEFV Innate Immuity Regulator, Pyrin | -1.65817025 |
| PFKP | Phosphofructokinase, Platelet | 1.82738 |
| PBX4 | PBX Homeobox 4 | 1.838437 |
| SLC25A3 | Solute Carrier Family 25 Member 3 | 1.804969 |
| GALNT3 | Polypeptide N-Acetylgalactosaminyltransferase 3 | 2.2420535 |
| SUMO1 | Small Ubiquitin Like Modifier 1 | 1.66042025 |
| GP1BA | Glycoprotein Ib Platelet Subunit Alpha | -1.527067 |
| ZEB1 | Zinc Finger E-Box Binding Homeobox 1 | 1.73314725 |
| ERG | ETS Transcription Factor ERG | 1.662892 |
| FGR | FGR Proto-Oncogene, Src Family Tyrosine Kinase | 1.65467325 |
| MET | MET Proto-Oncogene, Receptor Tyrosine Kinase | 1.7652735 |
| MAP3K3 | Mitogen-Activated Protein Kinase Kinase Kinase 3 | 1.9180345 |
| IGSF3 | Immunoglobulin Superfamily Member 3 | -2.77895 |
| SORL1 | Sortilin Related Receptor 1 | -2.43630125 |
| SERPINF1 | Serpin Family F Member 1 | 2.374697 |
| SLK | STE20 Like Kinase | 1.868363 |
| A2M | Alpha-2-Macroglobulin | -2.90731575 |
| ROCK2 | Rho Associated Coiled-Coil Containing Protein Kinase 2 | 1.60200225 |
| COL1A1 | Collagen Type I Alpha 1 Chain | 5.7744295 |
| NCOR2 | Nuclear Receptor Corepressor 2 | 1.5573615 |
| APOE | Apolipoprotein E | -2.7587335 |
| GRB2 | Growth Factor Receptor Bound Protein 2 | 1.97876225 |
| LCOR | Ligand Dependent Nuclear Receptor Corepressor | 2.09976675 |
| RPS28 | Ribosomal Protein S28 | 1.57384875 |
| GCLM | Glutamate-Cysteine Ligase Modifier Subunit | 1.817985 |
| ARL13B | ADP Ribosylation Factor Like GTPase 13B | 1.96868175 |
| BDKRB1 | Bradykinin Receptor B1 | 3.26632425 |
| DUSP5 | Dual Specificity Phosphatase 5 | 1.52942125 |
| COL18A1 | Collagen Type XVIII Alpha 1 Chain | 2.27754325 |
| MMP15 | Matrix Metallopeptidase 15 | -3.74228075 |
| F7 | Coagulation Factor VII | -2.98230575 |
| TFR2 | Transferrin Receptor 2 | -1.819244 |
| POMT1 | Protein O-Mannosyltransferase 1 | 1.885418 |
| KDM6A | Lysine Demethylase 6A | 1.68586275 |
| GLIS2 | GLIS Family Zinc Finger 2 | 2.0113705 |
| NFIL3 | Nuclear Factor, Interleukin 3 Regulated | 3.5375465 |
| CD53 | CD53 Molecule | -1.5215565 |
| PCCB | Propionyl-CoA Carboxylase Subunit Beta | 1.567885 |
| NADSYN1 | NAD Synthetase 1 | -1.7482285 |
| YIPF2 | Yip1 Domain Family Member 2 | 1.521346 |
| IL17C | Interleukin 17C | -2.79841525 |
| CHMP2B | Charged Multivesicular Body Protein 2B | 2.13608375 |
| KLF4 | KLF Transcription Factor 4 | 1.6504945 |
| SKAP2 | Src Kinase Associated Phosphoprotein 2 | 3.1557475 |
| TRAF7 | TNF Receptor Associated Factor 7 | 2.561062 |
| RAB38 | RAB38, Member RAS Oncogene Family | 1.73062575 |
| TNFAIP6 | TNF Alpha Induced Protein 6 | 4.32808375 |
| CD151 | CD151 Molecule (Raph Blood Group) | 1.7687425 |
| SOX9 | SRY-Box Transcription Factor 9 | 1.900371 |
| CPVL | Carboxypeptidase Vitellogenic Like | -2.0940655 |
| LDLR | Low Density Lipoprotein Receptor | 2.0599325 |
| KLF2 | KLF Transcription Factor 2 | 4.2733275 |
| DCT | Dopachrome Tautomerase | -3.3383485 |
| HLA-DOA | Major Histocompatibility Complex, Class II, DO Alpha | -2.166422 |
| RCAN1 | Regulator Of Calcineurin 1 | 2.8315355 |
| EIF2S1 | Eukaryotic Translation Initiation Factor 2 Subunit Alpha | 1.6013835 |
| RBM7 | RNA Binding Motif Protein 7 | 2.053368 |
| CD177 | CD177 Molecule | -1.6369315 |
| JAK2 | Janus Kinase 2 | 2.44960675 |
| RAB5A | RAB5A, Member RAS Oncogene Family | 1.71990925 |
| CA9 | Carbonic Anhydrase 9 | 2.42249775 |
| NEGR1 | Neuronal Growth Regulator 1 | -1.61086025 |
| BCL7A | BAF Chromatin Remodeling Complex Subunit BCL7A | 1.54920425 |
| MICA | MHC Class I Polypeptide-Related Sequence A | 1.99185325 |
| NT5E | 5'-Nucleotidase Ecto | 3.13949525 |
| NCSTN | Nicastrin | 1.57080475 |
| IGFBP2 | Insulin Like Growth Factor Binding Protein 2 | -1.98453375 |
| SMS | Spermine Synthase | 1.55241325 |
| FLI1 | Fli-1 Proto-Oncogene, ETS Transcription Factor | 1.735378 |
| TYMS | Thymidylate Synthetase | 2.50000475 |
| ALG9 | ALG9 Alpha-1,2-Mannosyltransferase | 1.50840675 |
| RABAC1 | Rab Acceptor 1 | 1.87910475 |
| MEF2B | Myocyte Enhancer Factor 2B | -1.98072975 |
| MAP2K4 | Mitogen-Activated Protein Kinase Kinase 4 | 1.7402055 |
| VEGFA | Vascular Endothelial Growth Factor A | 3.909346 |
| SOCS3 | Suppressor Of Cytokine Signaling 3 | 2.46551175 |
| ALPK1 | Alpha Kinase 1 | 1.83048025 |
| NKX2-1 | NK2 Homeobox 1 | -1.7365 |
| KIF5C | Kinesin Family Member 5C | 1.72960725 |
| MMP1 | Matrix Metallopeptidase 1 | 2.3920585 |
| HLA-DQA1 | Major Histocompatibility Complex, Class II, DQ Alpha 1 | -3.26019825 |
| TK2 | Thymidine Kinase 2 | 2.9445675 |
| CRNDE | Colorectal Neoplasia Differentially Expressed | 3.61794275 |
| RHBG | Rh Family B Glycoprotein | -1.555542 |
| TOR1A | Torsin Family 1 Member A | 1.56371975 |
| C4B | Complement C4B (Chido Blood Group) | -2.3861365 |
| OGFRL1 | Opioid Growth Factor Receptor Like 1 | 2.63997775 |
| CD1B | CD1b Molecule | -1.848214 |
| RPE | Ribulose-5-Phosphate-3-Epimerase | 1.62486175 |
| EIF4E | Eukaryotic Translation Initiation Factor 4E | 1.6084715 |
| ABHD5 | Abhydrolase Domain Containing 5, Lysophosphatidic Acid Acyltransferase | 1.9414855 |
| CCL18 | C-C Motif Chemokine Ligand 18 | -3.971468 |
| OGN | Osteoglycin | 3.56151725 |
| CXCR1 | C-X-C Motif Chemokine Receptor 1 | -2.464954 |
| BBS9 | Bardet-Biedl Syndrome 9 | 1.55012875 |
| PTGER2 | Prostaglandin E Receptor 2 | 2.56569125 |
| CEP290 | Centrosomal Protein 290 | 1.74420125 |
| CHD7 | Chromodomain Helicase DNA Binding Protein 7 | 2.61512625 |
| ALOX5AP | Arachidonate 5-Lipoxygenase Activating Protein | -1.9802625 |
| ANKH | ANKH Inorganic Pyrophosphate Transport Regulator | 1.931895 |
| ATP13A2 | ATPase Cation Transporting 13A2 | -3.064693 |
| EFHD2 | EF-Hand Domain Family Member D2 | 2.878851 |
| GNAI1 | G Protein Subunit Alpha I1 | 1.584745 |
| SFTA3 | Surfactant Associated 3 | -3.71227125 |
| TNPO1 | Transportin 1 | 1.91422 |
| PCNP | PEST Proteolytic Signal Containing Nuclear Protein | 1.51833 |
| CREB1 | CAMP Responsive Element Binding Protein 1 | 1.5885725 |
| CFL2 | Cofilin 2 | 2.21811325 |
| GIMAP4 | GTPase, IMAP Family Member 4 | -1.86701975 |
| STAT5B | Signal Transducer And Activator Of Transcription 5B | 1.95058025 |
| MFN1 | Mitofusin 1 | 1.823304 |
| MYOC | Myocilin | -2.15388175 |
| COMP | Cartilage Oligomeric Matrix Protein | 1.5942865 |
| APLN | Apelin | 1.85310975 |
| HRH1 | Histamine Receptor H1 | 1.7261845 |
| MCM4 | Minichromosome Maintenance Complex Component 4 | 1.61532625 |
| HTRA1 | HtrA Serine Peptidase 1 | 4.42000875 |
| BHLHE40 | Basic Helix-Loop-Helix Family Member E40 | 2.3255195 |
| P2RX1 | Purinergic Receptor P2X 1 | -2.89045525 |
| XRN1 | 5'-3' Exoribonuclease 1 | 1.87122975 |
| ADORA2B | Adenosine A2b Receptor | 3.111345 |
| MSMO1 | Methylsterol Monooxygenase 1 | 2.09598675 |
| SOX6 | SRY-Box Transcription Factor 6 | 1.527848 |
| POLE | DNA Polymerase Epsilon, Catalytic Subunit | -2.95609825 |
| KIF1A | Kinesin Family Member 1A | 1.7241415 |
| VCAN | Versican | 3.627868 |
| ARG2 | Arginase 2 | 1.720245 |
| SULF1 | Sulfatase 1 | 5.1375825 |
| SIGLEC5 | Sialic Acid Binding Ig Like Lectin 5 | -4.362694 |
| ZMYM2 | Zinc Finger MYM-Type Containing 2 | 1.54248675 |
| LPL | Lipoprotein Lipase | -1.6158215 |
| FAT1 | FAT Atypical Cadherin 1 | 1.80234775 |
| GJC1 | Gap Junction Protein Gamma 1 | 3.27725525 |
| SNHG12 | Small Nucleolar RNA Host Gene 12 | 1.71275025 |
| FPR2 | Formyl Peptide Receptor 2 | -1.72392 |
| BMI1 | BMI1 Proto-Oncogene, Polycomb Ring Finger | 1.6547495 |
| GNB1 | G Protein Subunit Beta 1 | 1.608055 |
| IGFBP3 | Insulin Like Growth Factor Binding Protein 3 | 1.92457025 |
| DEK | DEK Proto-Oncogene | 1.66060725 |
| COL11A1 | Collagen Type XI Alpha 1 Chain | 3.347224 |
| TREM1 | Triggering Receptor Expressed On Myeloid Cells 1 | 1.9319385 |
| MT2A | Metallothionein 2A | 2.0134615 |
| ROR1 | Receptor Tyrosine Kinase Like Orphan Receptor 1 | 2.203659 |
| SLC4A4 | Solute Carrier Family 4 Member 4 | -1.77697225 |
| COL1A2 | Collagen Type I Alpha 2 Chain | 2.74687825 |
| LY96 | Lymphocyte Antigen 96 | 1.913408 |
| PROCR | Protein C Receptor | 2.919733 |
| USP8 | Ubiquitin Specific Peptidase 8 | 1.55463325 |
| ITGB5 | Integrin Subunit Beta 5 | 2.1416515 |
| APOC1 | Apolipoprotein C1 | -2.2765895 |
| ARID1A | AT-Rich Interaction Domain 1A | 2.424547 |
| CYP4B1 | Cytochrome P450 Family 4 Subfamily B Member 1 | -2.20797675 |
| PNP | Purine Nucleoside Phosphorylase | 2.78191125 |
| ACTR2 | Actin Related Protein 2 | 2.0643235 |
| DARS2 | Aspartyl-TRNA Synthetase 2, Mitochondrial | 2.06068625 |
| KLF3 | KLF Transcription Factor 3 | 1.91039475 |
| PTGER3 | Prostaglandin E Receptor 3 | -1.529467 |
| LOC100289580 | Uncharacterized LOC100289580 | -4.77374075 |
| RYK | Receptor Like Tyrosine Kinase | 1.958953 |
| AIFM1 | Apoptosis Inducing Factor Mitochondria Associated 1 | 1.72004725 |
| MDFIC | MyoD Family Inhibitor Domain Containing | 1.93044825 |
| ADD3 | Adducin 3 | 2.48417625 |
| RAP1A | RAP1A, Member Of RAS Oncogene Family | 1.64484825 |
| NID1 | Nidogen 1 | 3.284346 |
| MMP13 | Matrix Metallopeptidase 13 | 6.5438195 |
| SLC25A37 | Solute Carrier Family 25 Member 37 | 3.9959145 |
| DSP | Desmoplakin | 4.409916 |
| PTK2B | Protein Tyrosine Kinase 2 Beta | 1.668819 |
| JAM3 | Junctional Adhesion Molecule 3 | 2.52216125 |
| TTF2 | Transcription Termination Factor 2 | 2.31059725 |
| SLC39A14 | Solute Carrier Family 39 Member 14 | 1.70267375 |
| CLN8 | CLN8 Transmembrane ER And ERGIC Protein | 2.05290825 |
| CRY2 | Cryptochrome Circadian Regulator 2 | 1.5545265 |
| NFATC4 | Nuclear Factor Of Activated T Cells 4 | 2.40358575 |
| BMPR1A | Bone Morphogenetic Protein Receptor Type 1A | 1.74820925 |
| CD55 | CD55 Molecule (Cromer Blood Group) | 2.60583775 |
| MDH1 | Malate Dehydrogenase 1 | 1.581358 |
| MYO6 | Myosin VI | 1.737488 |
| EZH2 | Enhancer Of Zeste 2 Polycomb Repressive Complex 2 Subunit | 2.04661375 |
| HBEGF | Heparin Binding EGF Like Growth Factor | 3.34035525 |
| PRKCI | Protein Kinase C Iota | 4.61165775 |
| POU2AF1 | POU Class 2 Homeobox Associating Factor 1 | 1.82500425 |
| TNFRSF11B | TNF Receptor Superfamily Member 11b | 1.989288 |
| PKLR | Pyruvate Kinase L/R | -2.68014675 |
| ARF4 | ADP Ribosylation Factor 4 | 2.026757 |
| NCF2 | Neutrophil Cytosolic Factor 2 | -1.7682125 |
| PIK3C3 | Phosphatidylinositol 3-Kinase Catalytic Subunit Type 3 | 2.08740375 |
| SOS2 | SOS Ras/Rho Guanine Nucleotide Exchange Factor 2 | 2.09082325 |
| ABO | ABO, Alpha 1-3-N-Acetylgalactosaminyltransferase And Alpha 1-3-Galactosyltransferase | -5.89308175 |
| CXCR2 | C-X-C Motif Chemokine Receptor 2 | -1.79765375 |
| SND1 | Staphylococcal Nuclease And Tudor Domain Containing 1 | 1.643909 |
| CS | Citrate Synthase | 2.047084 |
| IGFBP7 | Insulin Like Growth Factor Binding Protein 7 | 4.3432325 |
| FOLR1 | Folate Receptor Alpha | -2.01834575 |
| NEAT1 | Nuclear Paraspeckle Assembly Transcript 1 | 2.068966 |
| PRKCD | Protein Kinase C Delta | -1.95775425 |
| PABPC3 | Poly(A) Binding Protein Cytoplasmic 3 | 1.90246025 |
| CCL17 | C-C Motif Chemokine Ligand 17 | -1.5332445 |
| CD58 | CD58 Molecule | 2.90026275 |
| IREB2 | Iron Responsive Element Binding Protein 2 | 1.5498355 |
| THBD | Thrombomodulin | 2.6149225 |
| PIK3C2A | Phosphatidylinositol-4-Phosphate 3-Kinase Catalytic Subunit Type 2 Alpha | -1.714759 |
| NOL3 | Nucleolar Protein 3 | 1.68495175 |
| LTBP2 | Latent Transforming Growth Factor Beta Binding Protein 2 | -1.5102955 |
| UBR5 | Ubiquitin Protein Ligase E3 Component N-Recognin 5 | 1.585553 |
| LEF1 | Lymphoid Enhancer Binding Factor 1 | 4.7927205 |
| KCNQ1OT1 | KCNQ1 Opposite Strand/Antisense Transcript 1 | 1.84120025 |
| PITX2 | Paired Like Homeodomain 2 | 2.71568625 |
| SATB2 | SATB Homeobox 2 | 1.56270925 |
| MORC3 | MORC Family CW-Type Zinc Finger 3 | 2.14383725 |
| ALG13 | ALG13 UDP-N-Acetylglucosaminyltransferase Subunit | 2.1939475 |
| SMARCA5 | SWI/SNF Related, Matrix Associated, Actin Dependent Regulator Of Chromatin, Subfamily A, Member 5 | 1.543302 |
| PLA2G4A | Phospholipase A2 Group IVA | 2.90811025 |
| VEGFC | Vascular Endothelial Growth Factor C | 4.8825625 |
| VIM | Vimentin | 1.67174575 |
| HLA-DMB | Major Histocompatibility Complex, Class II, DM Beta | -2.96062 |
| ADAMTS4 | ADAM Metallopeptidase With Thrombospondin Type 1 Motif 4 | 1.8833535 |
| SCD | Stearoyl-CoA Desaturase | 1.530022 |
| PEX13 | Peroxisomal Biogenesis Factor 13 | 1.6267555 |
| EGLN1 | Egl-9 Family Hypoxia Inducible Factor 1 | 2.027002 |
| STX7 | Syntaxin 7 | 2.3760115 |
| H19 | H19 Imprinted Maternally Expressed Transcript | 2.618628 |
| TGFBR1 | Transforming Growth Factor Beta Receptor 1 | 2.4095175 |
| TNKS | Tankyrase | 1.6836785 |
| RBX1 | Ring-Box 1 | 2.09719325 |
| PTX3 | Pentraxin 3 | 2.4632645 |
| SMAD4 | SMAD Family Member 4 | 1.6011635 |
| PFKM | Phosphofructokinase, Muscle | 1.6990565 |
| MLLT3 | MLLT3 Super Elongation Complex Subunit | 1.53895925 |
| UBE2B | Ubiquitin Conjugating Enzyme E2 B | 2.537983 |
| LEMD3 | LEM Domain Containing 3 | 2.09495825 |
| AIF1 | Allograft Inflammatory Factor 1 | -1.6228 |
| SOAT1 | Sterol O-Acyltransferase 1 | 2.6156235 |
| ACHE | Acetylcholinesterase (Cartwright Blood Group) | -1.874525 |
| MCL1 | MCL1 Apoptosis Regulator, BCL2 Family Member | 1.63395675 |
| PTBP1 | Polypyrimidine Tract Binding Protein 1 | 2.7585135 |
| MVP | Major Vault Protein | 2.11987475 |
| TOPORS | TOP1 Binding Arginine/Serine Rich Protein, E3 Ubiquitin Ligase | 1.90145875 |
| XPR1 | Xenotropic And Polytropic Retrovirus Receptor 1 | 2.1560685 |
| ITGAM | Integrin Subunit Alpha M | -2.72535625 |
| RGS2 | Regulator Of G Protein Signaling 2 | 3.14533775 |
| RTN4 | Reticulon 4 | 1.69653175 |
| COL3A1 | Collagen Type III Alpha 1 Chain | 3.875739 |
| CD163 | CD163 Molecule | -2.63890775 |
| WDR11 | WD Repeat Domain 11 | 1.80637325 |
| CDK9 | Cyclin Dependent Kinase 9 | 2.32384 |
| CD59 | CD59 Molecule (CD59 Blood Group) | 2.1039845 |
| BST2 | Bone Marrow Stromal Cell Antigen 2 | -2.448324 |
| BMPR2 | Bone Morphogenetic Protein Receptor Type 2 | 2.32824325 |
| SERPINE2 | Serpin Family E Member 2 | 4.6646545 |
| ADAM33 | ADAM Metallopeptidase Domain 33 | -1.93048625 |
| NUBPL | NUBP Iron-Sulfur Cluster Assembly Factor, Mitochondrial | 2.0511895 |
| ID4 | Inhibitor Of DNA Binding 4, HLH Protein | 2.83996425 |
| PIGA | Phosphatidylinositol Glycan Anchor Biosynthesis Class A | 2.44714325 |
| C8B | Complement C8 Beta Chain | 2.148016 |
| SP1 | Sp1 Transcription Factor | 1.955892 |
| PLAG1 | PLAG1 Zinc Finger | 1.72012975 |
| PUS7 | Pseudouridine Synthase 7 | 1.513616 |
| FGF2 | Fibroblast Growth Factor 2 | 3.84234975 |
| COMMD10 | COMM Domain Containing 10 | 1.675031 |
| GPSM3 | G Protein Signaling Modulator 3 | -2.85309775 |
| SPTB | Spectrin Beta, Erythrocytic | -1.7267305 |
| CAV1 | Caveolin 1 | 2.01021775 |
| PIGN | Phosphatidylinositol Glycan Anchor Biosynthesis Class N | 2.2671825 |
| PRSS23 | Serine Protease 23 | 4.282421 |
| ABCB4 | ATP Binding Cassette Subfamily B Member 4 | 2.70301075 |
| SFTPB | Surfactant Protein B | -3.4096845 |
| FBL | Fibrillarin | 1.56845325 |
| LOXL3 | Lysyl Oxidase Like 3 | 2.62541925 |
| PKN1 | Protein Kinase N1 | 1.63823825 |
| RHBDF2 | Rhomboid 5 Homolog 2 | 2.6607685 |
| EIF2AK4 | Eukaryotic Translation Initiation Factor 2 Alpha Kinase 4 | 1.70061225 |
| AQP1 | Aquaporin 1 (Colton Blood Group) | 2.5511465 |
| ANKRD12 | Ankyrin Repeat Domain 12 | 1.6832005 |
| NOX1 | NADPH Oxidase 1 | -2.56667825 |
| GLIPR1 | GLI Pathogenesis Related 1 | 1.5041715 |
| HLA-DRB1 | Major Histocompatibility Complex, Class II, DR Beta 1 | -3.0550125 |
| SLC35A3 | Solute Carrier Family 35 Member A3 | 1.54117825 |
| PAICS | Phosphoribosylaminoimidazole Carboxylase And Phosphoribosylaminoimidazolesuccinocarboxamide Synthase | 2.45104375 |
| GPX5 | Glutathione Peroxidase 5 | 1.68131275 |
| VANGL1 | VANGL Planar Cell Polarity Protein 1 | 1.50337075 |
| CANX | Calnexin | 1.9919 |
| FRZB | Frizzled Related Protein | -5.9761755 |
| PABPC1 | Poly(A) Binding Protein Cytoplasmic 1 | 1.67681475 |
| COL6A3 | Collagen Type VI Alpha 3 Chain | 2.1455935 |
| TMEM126B | Transmembrane Protein 126B | 1.97528375 |
| NDUFA9 | NADH:Ubiquinone Oxidoreductase Subunit A9 | 2.17310775 |
| EGLN3 | Egl-9 Family Hypoxia Inducible Factor 3 | 3.81145025 |
| SLC25A12 | Solute Carrier Family 25 Member 12 | 2.8219095 |
| HPX | Hemopexin | -2.34723075 |
| TIMP2 | TIMP Metallopeptidase Inhibitor 2 | 1.79203925 |
| SAE1 | SUMO1 Activating Enzyme Subunit 1 | 1.64275825 |
| COL6A1 | Collagen Type VI Alpha 1 Chain | 3.3659915 |
| SGCE | Sarcoglycan Epsilon | 1.866975 |
| IL11 | Interleukin 11 | 5.53757025 |
| KITLG | KIT Ligand | -1.59889925 |
| SETX | Senataxin | 2.491467 |
| MIR22HG | MIR22 Host Gene | -2.30188675 |
| SPARC | Secreted Protein Acidic And Cysteine Rich | 1.567584 |
| KCTD12 | Potassium Channel Tetramerization Domain Containing 12 | 2.04245675 |
| WWP2 | WW Domain Containing E3 Ubiquitin Protein Ligase 2 | 1.5769235 |
| CTSH | Cathepsin H | -3.5698655 |
| COL5A1 | Collagen Type V Alpha 1 Chain | 2.38967625 |
| IFT20 | Intraflagellar Transport 20 | 1.981808 |
| RELN | Reelin | -2.98369 |
| HAS1 | Hyaluronan Synthase 1 | 1.669261 |
| TRPS1 | Transcriptional Repressor GATA Binding 1 | 2.22652725 |
| PDIA4 | Protein Disulfide Isomerase Family A Member 4 | 1.60161975 |
| JUN | Jun Proto-Oncogene, AP-1 Transcription Factor Subunit | 2.924525 |
| NOTCH3 | Notch Receptor 3 | 2.96077125 |
| TRIM25 | Tripartite Motif Containing 25 | 1.8341715 |
| CRKL | CRK Like Proto-Oncogene, Adaptor Protein | 2.30619325 |
| ZNF133 | Zinc Finger Protein 133 | -2.16212325 |
| ADCY9 | Adenylate Cyclase 9 | 1.812361 |
| TMF1 | TATA Element Modulatory Factor 1 | 1.67015975 |
| TNFRSF10D | TNF Receptor Superfamily Member 10d | 3.0822595 |
| GSTT1 | Glutathione S-Transferase Theta 1 | 3.5627705 |
| USP1 | Ubiquitin Specific Peptidase 1 | 1.9198995 |
| SLC16A10 | Solute Carrier Family 16 Member 10 | 2.886169 |
| PTGIS | Prostaglandin I2 Synthase | 3.884461 |
| NDN | Necdin, MAGE Family Member | 2.29621075 |
| ITPR3 | Inositol 1,4,5-Trisphosphate Receptor Type 3 | 1.561219 |
| IFT52 | Intraflagellar Transport 52 | 2.01392125 |
| RMRP | RNA Component Of Mitochondrial RNA Processing Endoribonuclease | -1.61376075 |
| RBM15 | RNA Binding Motif Protein 15 | -1.5823 |
| NKX2-5 | NK2 Homeobox 5 | -2.738832 |
| GZMM | Granzyme M | -1.63980875 |
| HRG | Histidine Rich Glycoprotein | -3.1647105 |
| TRIM33 | Tripartite Motif Containing 33 | 2.1800645 |
| CEACAM6 | CEA Cell Adhesion Molecule 6 | -4.23541925 |
| ARF6 | ADP Ribosylation Factor 6 | 2.25130725 |
| FADS1 | Fatty Acid Desaturase 1 | 1.79441225 |
| CSF3R | Colony Stimulating Factor 3 Receptor | -2.5376955 |
| ELOVL5 | ELOVL Fatty Acid Elongase 5 | 1.54545175 |
| PAPSS2 | 3'-Phosphoadenosine 5'-Phosphosulfate Synthase 2 | 2.26953275 |
| SPTSSA | Serine Palmitoyltransferase Small Subunit A | 2.32160275 |
| DYSF | Dysferlin | 3.206702 |
| LAIR1 | Leukocyte Associated Immunoglobulin Like Receptor 1 | -2.132469 |
| RBPJ | Recombination Signal Binding Protein For Immunoglobulin Kappa J Region | 2.25974725 |
| SLCO2A1 | Solute Carrier Organic Anion Transporter Family Member 2A1 | -3.24906425 |
| PRDM16 | PR/SET Domain 16 | -1.6912015 |
| NDUFA8 | NADH:Ubiquinone Oxidoreductase Subunit A8 | 1.5951035 |
| SORT1 | Sortilin 1 | 2.7683425 |
| KIR2DL4 | Killer Cell Immunoglobulin Like Receptor, Two Ig Domains And Long Cytoplasmic Tail 4 | -2.33074175 |
| MCU | Mitochondrial Calcium Uniporter | 1.89697125 |
| MAFK | MAF BZIP Transcription Factor K | 1.64406825 |
| RACGAP1 | Rac GTPase Activating Protein 1 | 2.641317 |
| AVEN | Apoptosis And Caspase Activation Inhibitor | 1.57279025 |
| HLA-DRA | Major Histocompatibility Complex, Class II, DR Alpha | -4.46552425 |
| ADAMTS16 | ADAM Metallopeptidase With Thrombospondin Type 1 Motif 16 | 1.56450825 |
| FUCA2 | Alpha-L-Fucosidase 2 | 1.84647525 |
| CRIPT | CXXC Repeat Containing Interactor Of PDZ3 Domain | 1.622487 |
| LTBP1 | Latent Transforming Growth Factor Beta Binding Protein 1 | 2.90188725 |
| BACE2 | Beta-Secretase 2 | 2.375555 |
| EZR | Ezrin | 1.82315025 |
| ATF1 | Activating Transcription Factor 1 | 1.6654465 |
| PTGES | Prostaglandin E Synthase | 2.3895155 |
| TP73-AS1 | TP73 Antisense RNA 1 | 1.7970205 |
| CHI3L1 | Chitinase 3 Like 1 | -1.7200725 |
| CLEC3B | C-Type Lectin Domain Family 3 Member B | 5.8583325 |
| OCRL | OCRL Inositol Polyphosphate-5-Phosphatase | 1.52389975 |
| RARB | Retinoic Acid Receptor Beta | 1.5085795 |
| AGGF1 | Angiogenic Factor With G-Patch And FHA Domains 1 | 2.23244325 |
| HLF | HLF Transcription Factor, PAR BZIP Family Member | 2.13650575 |
| FRYL | FRY Like Transcription Coactivator | 2.627619 |
| AGTRAP | Angiotensin II Receptor Associated Protein | 1.576943 |
| TIAM1 | TIAM Rac1 Associated GEF 1 | 3.1562655 |
| FN1 | Fibronectin 1 | 3.6059715 |
| GANC | Glucosidase Alpha, Neutral C | 2.95821075 |
| PPP1R12A | Protein Phosphatase 1 Regulatory Subunit 12A | 2.1780035 |
| TPMT | Thiopurine S-Methyltransferase | 2.0261145 |
| LOXL1 | Lysyl Oxidase Like 1 | 1.74141525 |
| NR1I3 | Nuclear Receptor Subfamily 1 Group I Member 3 | -1.65836875 |
| WNT3A | Wnt Family Member 3A | -2.32288 |
| CSTA | Cystatin A | -2.08346725 |
| BMP6 | Bone Morphogenetic Protein 6 | 2.951111 |
| LAMB3 | Laminin Subunit Beta 3 | 1.607612 |
| KCTD10 | Potassium Channel Tetramerization Domain Containing 10 | 1.533069 |
| TNF | Tumor Necrosis Factor | -1.51255725 |
| ASAP1 | ArfGAP With SH3 Domain, Ankyrin Repeat And PH Domain 1 | 2.098009 |
| PDGFD | Platelet Derived Growth Factor D | 2.18056025 |
| CREBBP | CREB Binding Protein | 2.419907 |
| ITGB1 | Integrin Subunit Beta 1 | 1.8039005 |
| SGK1 | Serum/Glucocorticoid Regulated Kinase 1 | 3.46641675 |
| COL5A2 | Collagen Type V Alpha 2 Chain | 3.1855405 |
| NOP10 | NOP10 Ribonucleoprotein | -1.667469 |
| XRCC4 | X-Ray Repair Cross Complementing 4 | 2.27809725 |
| THY1 | Thy-1 Cell Surface Antigen | 3.863125 |
| GLI1 | GLI Family Zinc Finger 1 | 2.583901 |
| SPP1 | Secreted Phosphoprotein 1 | 4.01934375 |
| SFTPD | Surfactant Protein D | -1.94207975 |
| GNS | Glucosamine (N-Acetyl)-6-Sulfatase | 2.08985125 |
| ENO2 | Enolase 2 | 2.3045895 |
| PTGS2 | Prostaglandin-Endoperoxide Synthase 2 | 3.63876525 |
| WBP11 | WW Domain Binding Protein 11 | 1.65917225 |
| TMEM176A | Transmembrane Protein 176A | -2.04223775 |
| GALNT2 | Polypeptide N-Acetylgalactosaminyltransferase 2 | 2.0611245 |
| HAS2 | Hyaluronan Synthase 2 | 2.184532 |
| FCGR3A | Fc Gamma Receptor IIIa | -2.020042 |
| C1GALT1 | Core 1 Synthase, Glycoprotein-N-Acetylgalactosamine 3-Beta-Galactosyltransferase 1 | 1.62463475 |
| CCND1 | Cyclin D1 | 2.85640975 |
| USP18 | Ubiquitin Specific Peptidase 18 | -3.1402195 |
| BMP2 | Bone Morphogenetic Protein 2 | 2.64148125 |
| LST1 | Leukocyte Specific Transcript 1 | -1.68778325 |
| FMOD | Fibromodulin | 1.74398475 |
| XYLT2 | Xylosyltransferase 2 | 1.65221925 |
| CRAT | Carnitine O-Acetyltransferase | 2.199228 |
| SLC19A1 | Solute Carrier Family 19 Member 1 | -2.85429375 |
| UBE2L3 | Ubiquitin Conjugating Enzyme E2 L3 | 1.748028 |
| BIRC2 | Baculoviral IAP Repeat Containing 2 | 1.87043825 |
| PLS3 | Plastin 3 | 2.646407 |
| PLOD2 | Procollagen-Lysine,2-Oxoglutarate 5-Dioxygenase 2 | 3.169146 |
| PI3 | Peptidase Inhibitor 3 | -1.765316 |
| CTSF | Cathepsin F | 1.72084675 |
| POU2F2 | POU Class 2 Homeobox 2 | -1.58594325 |
| STAT1 | Signal Transducer And Activator Of Transcription 1 | 1.79558825 |
| UBE2T | Ubiquitin Conjugating Enzyme E2 T | 1.800572 |
| SLC2A1 | Solute Carrier Family 2 Member 1 | 1.83916575 |
| CD68 | CD68 Molecule | 1.73908425 |
| SMARCA1 | SWI/SNF Related, Matrix Associated, Actin Dependent Regulator Of Chromatin, Subfamily A, Member 1 | 1.7092075 |
| WEE1 | WEE1 G2 Checkpoint Kinase | 2.302684 |
| NR2F2 | Nuclear Receptor Subfamily 2 Group F Member 2 | 1.97526975 |
| PLXND1 | Plexin D1 | 1.64201025 |
| BSG | Basigin (Ok Blood Group) | 2.06632125 |
| NAP1L1 | Nucleosome Assembly Protein 1 Like 1 | 1.9079225 |
| CD14 | CD14 Molecule | 1.51031925 |
| HMGCR | 3-Hydroxy-3-Methylglutaryl-CoA Reductase | 1.87610875 |
| GGH | Gamma-Glutamyl Hydrolase | 3.569106 |
| GGT1 | Gamma-Glutamyltransferase 1 | -1.85678 |
| LUM | Lumican | 2.429796 |
| UBASH3B | Ubiquitin Associated And SH3 Domain Containing B | 2.97900925 |
| FEM1C | Fem-1 Homolog C | 2.35297025 |
| U2AF2 | U2 Small Nuclear RNA Auxiliary Factor 2 | 1.5614325 |
| SMAD2 | SMAD Family Member 2 | 2.3204305 |
| PLD2 | Phospholipase D2 | 1.82807575 |
| PPP3CB | Protein Phosphatase 3 Catalytic Subunit Beta | 1.7673025 |
| ANGPTL4 | Angiopoietin Like 4 | 4.70195525 |
| TEP1 | Telomerase Associated Protein 1 | -1.58410225 |
| KIDINS220 | Kinase D Interacting Substrate 220 | 1.52757325 |
| GADD45A | Growth Arrest And DNA Damage Inducible Alpha | 1.6610315 |
| POSTN | Periostin | 5.72503775 |
| PLA2G1B | Phospholipase A2 Group IB | -2.00438275 |
| PLAT | Plasminogen Activator, Tissue Type | 3.9408095 |
| FRG1 | FSHD Region Gene 1 | 2.50247175 |
| MUC6 | Mucin 6, Oligomeric Mucus/Gel-Forming | -2.8463265 |
| MYLK | Myosin Light Chain Kinase | -2.76823 |
| DOK1 | Docking Protein 1 | 2.48929025 |
| ASPG | Asparaginase | -2.23306575 |
| SRD5A1 | Steroid 5 Alpha-Reductase 1 | 1.89263125 |
| IL17F | Interleukin 17F | -1.6700625 |
| SELPLG | Selectin P Ligand | -2.863933 |
| TTC8 | Tetratricopeptide Repeat Domain 8 | 1.67226575 |
| XIST | X Inactive Specific Transcript | -1.59810475 |
| CCNT1 | Cyclin T1 | -2.3974895 |
| APOD | Apolipoprotein D | -3.870414 |
| SYNCRIP | Synaptotagmin Binding Cytoplasmic RNA Interacting Protein | 1.74871825 |
| ACVR1 | Activin A Receptor Type 1 | 1.8737285 |
| RNASEH2B | Ribonuclease H2 Subunit B | 1.93762975 |
| HLA-DQA2 | Major Histocompatibility Complex, Class II, DQ Alpha 2 | -1.8815155 |
| PIK3R2 | Phosphoinositide-3-Kinase Regulatory Subunit 2 | 2.34319175 |
| EBF2 | EBF Transcription Factor 2 | -2.53573675 |
| MTDH | Metadherin | 1.530611 |
| EIF4EBP1 | Eukaryotic Translation Initiation Factor 4E Binding Protein 1 | -2.073785 |
| SUZ12 | SUZ12 Polycomb Repressive Complex 2 Subunit | 2.1867745 |
| SOCS1 | Suppressor Of Cytokine Signaling 1 | -1.58224675 |
| IL17RA | Interleukin 17 Receptor A | 1.53987525 |
| GGCX | Gamma-Glutamyl Carboxylase | 1.672756 |
| RB1 | RB Transcriptional Corepressor 1 | 2.0330875 |
| BZW2 | Basic Leucine Zipper And W2 Domains 2 | 1.9882625 |
| CELF2 | CUGBP Elav-Like Family Member 2 | 1.6892255 |
| SAA1 | Serum Amyloid A1 | -3.600481 |
| FBXW7 | F-Box And WD Repeat Domain Containing 7 | 2.143754 |
| NUP107 | Nucleoporin 107 | 1.57887825 |
| TIMELESS | Timeless Circadian Regulator | 1.9574875 |
| MAPRE2 | Microtubule Associated Protein RP/EB Family Member 2 | 1.85794775 |
| ARFGAP3 | ADP Ribosylation Factor GTPase Activating Protein 3 | 1.60892875 |
| KRT7 | Keratin 7 | -2.1105985 |
| SGCD | Sarcoglycan Delta | 1.738459 |
| KANK2 | KN Motif And Ankyrin Repeat Domains 2 | 1.7110945 |
| EMILIN2 | Elastin Microfibril Interfacer 2 | 1.7210895 |
| STK11 | Serine/Threonine Kinase 11 | 2.25847425 |
| VASP | Vasodilator Stimulated Phosphoprotein | 2.12915375 |
| LOX | Lysyl Oxidase | 3.60541225 |
| UMOD | Uromodulin | -2.08469075 |
| ZNF469 | Zinc Finger Protein 469 | 1.647971 |
| BRD2 | Bromodomain Containing 2 | 1.69832425 |
| IL32 | Interleukin 32 | -1.6379685 |
| CPOX | Coproporphyrinogen Oxidase | 2.23043225 |
| F5 | Coagulation Factor V | 2.01054125 |
| ABCA2 | ATP Binding Cassette Subfamily A Member 2 | -1.55009025 |
| CDKN2C | Cyclin Dependent Kinase Inhibitor 2C | 2.04810075 |
| ADM | Adrenomedullin | 3.9183975 |
| MAPK8IP3 | Mitogen-Activated Protein Kinase 8 Interacting Protein 3 | 1.67507 |
| ABCC1 | ATP Binding Cassette Subfamily C Member 1 | 1.6086075 |
| SBSN | Suprabasin | 3.292965 |
| SIAE | Sialic Acid Acetylesterase | 1.87315225 |
| SHOX2 | Short Stature Homeobox 2 | -2.86665325 |
| PLVAP | Plasmalemma Vesicle Associated Protein | -1.69464475 |
| POU2F1 | POU Class 2 Homeobox 1 | 2.21604875 |
| MLLT11 | MLLT11 Transcription Factor 7 Cofactor | 1.69065075 |
| TCN2 | Transcobalamin 2 | -1.59908425 |
| ACTG1 | Actin Gamma 1 | 1.7058745 |
| KDSR | 3-Ketodihydrosphingosine Reductase | 1.6083095 |
| LACTB | Lactamase Beta | 1.61975875 |
| SIK1 | Salt Inducible Kinase 1 | 1.78990725 |
| SLC16A1 | Solute Carrier Family 16 Member 1 | 1.83440025 |
| LNX1 | Ligand Of Numb-Protein X 1 | 2.445484 |
| TRADD | TNFRSF1A Associated Via Death Domain | 1.632531 |
| SLC19A2 | Solute Carrier Family 19 Member 2 | 2.15126575 |
| B3GNT2 | UDP-GlcNAc:BetaGal Beta-1,3-N-Acetylglucosaminyltransferase 2 | 2.6519905 |
| S1PR3 | Sphingosine-1-Phosphate Receptor 3 | 3.34436975 |
| PDE3B | Phosphodiesterase 3B | -1.8517855 |
| STEAP1 | STEAP Family Member 1 | 2.910589 |
| PGK1 | Phosphoglycerate Kinase 1 | 1.96735025 |
| OAT | Ornithine Aminotransferase | 1.53139725 |
| TLX3 | T Cell Leukemia Homeobox 3 | -2.4167185 |
| PTEN | Phosphatase And Tensin Homolog | 1.5363725 |
| ANXA1 | Annexin A1 | 1.9334835 |
| DCK | Deoxycytidine Kinase | 1.67796275 |
| TIA1 | TIA1 Cytotoxic Granule Associated RNA Binding Protein | 2.15611725 |
| DNAJB1 | DnaJ Heat Shock Protein Family (Hsp40) Member B1 | 1.8864965 |
| GJA1 | Gap Junction Protein Alpha 1 | 3.853603 |
| GNAI2 | G Protein Subunit Alpha I2 | 1.85321875 |
| RAB3GAP1 | RAB3 GTPase Activating Protein Catalytic Subunit 1 | 1.55597925 |
| CLDN4 | Claudin 4 | -1.94390075 |
| UCHL1 | Ubiquitin C-Terminal Hydrolase L1 | 3.4253825 |
| PHF3 | PHD Finger Protein 3 | 1.8282615 |
| DICER1 | Dicer 1, Ribonuclease III | 1.7713435 |
| LGALS1 | Galectin 1 | 2.1012225 |
| NEUROG3 | Neurogenin 3 | -2.79574475 |
| LMBRD1 | LMBR1 Domain Containing 1 | 1.99312125 |
| SEMA3A | Semaphorin 3A | 2.20596975 |
| BTRC | Beta-Transducin Repeat Containing E3 Ubiquitin Protein Ligase | 2.067237 |
| CSF1 | Colony Stimulating Factor 1 | -1.5987975 |
| THBS3 | Thrombospondin 3 | 1.75140225 |
| SLC2A3 | Solute Carrier Family 2 Member 3 | 2.855273 |
| C3AR1 | Complement C3a Receptor 1 | -2.424526 |
| BDKRB2 | Bradykinin Receptor B2 | 2.00897 |
| GPI | Glucose-6-Phosphate Isomerase | 1.57326925 |
| RAD9A | RAD9 Checkpoint Clamp Component A | 1.57147475 |
| RAI14 | Retinoic Acid Induced 14 | 1.81985925 |
| TSC22D3 | TSC22 Domain Family Member 3 | 2.69201125 |
| CFH | Complement Factor H | -1.66395175 |
| MOG | Myelin Oligodendrocyte Glycoprotein | -1.7923185 |
| BBS10 | Bardet-Biedl Syndrome 10 | 1.7744815 |
| APLNR | Apelin Receptor | -1.663783 |
| JAG1 | Jagged Canonical Notch Ligand 1 | 1.902999 |
| MAPT | Microtubule Associated Protein Tau | 1.6389615 |
| NAGA | Alpha-N-Acetylgalactosaminidase | 1.63123975 |
| HLA-DQB1 | Major Histocompatibility Complex, Class II, DQ Beta 1 | -2.6995295 |
| MNDA | Myeloid Cell Nuclear Differentiation Antigen | -2.2025795 |
| CRY1 | Cryptochrome Circadian Regulator 1 | 2.6784345 |
| PIM1 | Pim-1 Proto-Oncogene, Serine/Threonine Kinase | 2.63599575 |
| USP9X | Ubiquitin Specific Peptidase 9 X-Linked | 1.70382375 |
| AOC3 | Amine Oxidase Copper Containing 3 | 1.88173575 |
| FAF1 | Fas Associated Factor 1 | 1.9994675 |
| CDK6 | Cyclin Dependent Kinase 6 | 3.93042425 |
| RAB27B | RAB27B, Member RAS Oncogene Family | -1.6065195 |
| LYST | Lysosomal Trafficking Regulator | 1.8912975 |
| NCR3 | Natural Cytotoxicity Triggering Receptor 3 | -1.93375625 |
| HLA-DPA1 | Major Histocompatibility Complex, Class II, DP Alpha 1 | -1.66577025 |
| APOA5 | Apolipoprotein A5 | -1.81061225 |
| ITGAV | Integrin Subunit Alpha V | 1.59655775 |
| REV3L | REV3 Like, DNA Directed Polymerase Zeta Catalytic Subunit | 1.6003645 |
| PRKAR1A | Protein Kinase CAMP-Dependent Type I Regulatory Subunit Alpha | 2.23122225 |
| ATG5 | Autophagy Related 5 | 1.92025175 |
| CD2AP | CD2 Associated Protein | 1.719232 |
| CWC27 | CWC27 Spliceosome Associated Cyclophilin | 2.75045425 |
| SPRED1 | Sprouty Related EVH1 Domain Containing 1 | 1.88216 |
| HBD | Hemoglobin Subunit Delta | 1.598491 |
| IRAK3 | Interleukin 1 Receptor Associated Kinase 3 | 1.8908205 |
| ADAM10 | ADAM Metallopeptidase Domain 10 | 2.5999805 |
| GALC | Galactosylceramidase | -2.56991625 |
| GAMT | Guanidinoacetate N-Methyltransferase | -1.96557425 |
| CYP2E1 | Cytochrome P450 Family 2 Subfamily E Member 1 | -2.1050515 |
| ACO1 | Aconitase 1 | 1.56336675 |
| BMP4 | Bone Morphogenetic Protein 4 | 1.5088395 |
| TJP1 | Tight Junction Protein 1 | 2.5656075 |
| CD46 | CD46 Molecule | 1.55661425 |
| FKBP1B | FKBP Prolyl Isomerase 1B | 1.707744 |
| GLI3 | GLI Family Zinc Finger 3 | 2.54659675 |
| SLC17A7 | Solute Carrier Family 17 Member 7 | -1.736193 |
| MIAT | Myocardial Infarction Associated Transcript | 4.6557455 |
| FBN1 | Fibrillin 1 | 1.5804125 |
| CSNK1A1 | Casein Kinase 1 Alpha 1 | 2.5756235 |
| IQCB1 | IQ Motif Containing B1 | 3.06634125 |
| RAPGEF4 | Rap Guanine Nucleotide Exchange Factor 4 | 1.5111565 |
| EIF2AK3 | Eukaryotic Translation Initiation Factor 2 Alpha Kinase 3 | 2.07022825 |
| MED30 | Mediator Complex Subunit 30 | 1.7806665 |
| CCL24 | C-C Motif Chemokine Ligand 24 | -1.5047715 |
| ANGPT1 | Angiopoietin 1 | 2.206469 |
| EEF1A1 | Eukaryotic Translation Elongation Factor 1 Alpha 1 | 1.58127275 |
| NDUFC2 | NADH:Ubiquinone Oxidoreductase Subunit C2 | 1.63756025 |
| SLC37A4 | Solute Carrier Family 37 Member 4 | -1.81584 |
| TPSAB1 | Tryptase Alpha/Beta 1 | -1.8712855 |
| ENG | Endoglin | 1.51961025 |
| TK1 | Thymidine Kinase 1 | -2.71606275 |
| SNHG3 | Small Nucleolar RNA Host Gene 3 | 2.54678725 |
| KIR2DS4 | Killer Cell Immunoglobulin Like Receptor, Two Ig Domains And Short Cytoplasmic Tail 4 | -2.72936675 |
| KCNK2 | Potassium Two Pore Domain Channel Subfamily K Member 2 | 3.2294365 |
| GLUD1 | Glutamate Dehydrogenase 1 | 3.6406805 |
| MFAP4 | Microfibril Associated Protein 4 | 1.88479475 |
| NBEAL2 | Neurobeachin Like 2 | -1.516587 |
| TYROBP | Transmembrane Immune Signaling Adaptor TYROBP | -2.890112 |
| RARA | Retinoic Acid Receptor Alpha | -1.68036975 |
| TNC | Tenascin C | 1.84055525 |
| CBFB | Core-Binding Factor Subunit Beta | 1.6236555 |
| SERPINH1 | Serpin Family H Member 1 | 2.17142125 |
| RORA | RAR Related Orphan Receptor A | 2.98070075 |
| PTPRD | Protein Tyrosine Phosphatase Receptor Type D | 2.300324 |
| PRG4 | Proteoglycan 4 | 3.729034 |
| OPTN | Optineurin | 1.850314 |
| UGDH | UDP-Glucose 6-Dehydrogenase | 1.7760345 |
| ADPRH | ADP-Ribosylarginine Hydrolase | 1.707863 |
| DNMT1 | DNA Methyltransferase 1 | 2.203675 |
| PIWIL4 | Piwi Like RNA-Mediated Gene Silencing 4 | 1.688614 |
| P2RY6 | Pyrimidinergic Receptor P2Y6 | 1.6302265 |
| SNHG5 | Small Nucleolar RNA Host Gene 5 | 1.72186025 |
| LIFR | LIF Receptor Subunit Alpha | 1.54416625 |
| CBL | Cbl Proto-Oncogene | 1.8368945 |
| TSPO | Translocator Protein | 2.18624525 |
| VNN1 | Vanin 1 | -1.5136795 |
| CACNA1A | Calcium Voltage-Gated Channel Subunit Alpha1 A | 2.47566325 |
| ABI1 | Abl Interactor 1 | 1.675899 |
| CDH1 | Cadherin 1 | -2.18734175 |
| IFIT1 | Interferon Induced Protein With Tetratricopeptide Repeats 1 | -1.54776325 |
| GNAQ | G Protein Subunit Alpha Q | 1.89437875 |
| MMAA | Metabolism Of Cobalamin Associated A | 1.97078475 |
| SRSF1 | Serine And Arginine Rich Splicing Factor 1 | 3.34288225 |
| COL2A1 | Collagen Type II Alpha 1 Chain | 2.35197075 |
| IFI30 | IFI30 Lysosomal Thiol Reductase | -1.83126775 |
| SCARB2 | Scavenger Receptor Class B Member 2 | 1.93550225 |
| CHIC2 | Cysteine Rich Hydrophobic Domain 2 | 2.9048335 |
| SLC16A3 | Solute Carrier Family 16 Member 3 | 2.1331305 |
| IGLON5 | IgLON Family Member 5 | -3.88751325 |
| THBS2 | Thrombospondin 2 | 2.8675305 |
| TGOLN2 | Trans-Golgi Network Protein 2 | 1.8685925 |
| TRIM39 | Tripartite Motif Containing 39 | -1.6833345 |
| GRN | Granulin Precursor | 2.5255155 |
| AP3D1 | Adaptor Related Protein Complex 3 Subunit Delta 1 | 1.758112 |
| ANPEP | Alanyl Aminopeptidase, Membrane | 1.960507 |
| MSH2 | MutS Homolog 2 | 1.559475 |
| CYLD | CYLD Lysine 63 Deubiquitinase | 1.96681525 |
| JMJD1C | Jumonji Domain Containing 1C | 2.65917775 |
| USP48 | Ubiquitin Specific Peptidase 48 | 1.53126975 |
| ABCA4 | ATP Binding Cassette Subfamily A Member 4 | -2.00617775 |
| SMN1 | Survival Of Motor Neuron 1, Telomeric | 1.86410775 |
| VCAM1 | Vascular Cell Adhesion Molecule 1 | 1.96732925 |
| STAT2 | Signal Transducer And Activator Of Transcription 2 | 1.52044175 |
| PTAFR | Platelet Activating Factor Receptor | -2.0389465 |
| EWSR1 | EWS RNA Binding Protein 1 | 1.525281 |
| AKAP13 | A-Kinase Anchoring Protein 13 | 2.0748845 |
| ANGPTL2 | Angiopoietin Like 2 | 2.371684 |
| WFDC3 | WAP Four-Disulfide Core Domain 3 | -1.90137 |
| CRK | CRK Proto-Oncogene, Adaptor Protein | 1.5146105 |
| HSPA1B | Heat Shock Protein Family A (Hsp70) Member 1B | 2.9293695 |
| RGS10 | Regulator Of G Protein Signaling 10 | 1.50812125 |
| CCND2 | Cyclin D2 | 2.801821 |
| NPTX1 | Neuronal Pentraxin 1 | -1.50556975 |
| CLEC4G | C-Type Lectin Domain Family 4 Member G | -1.8320105 |
| RAD21 | RAD21 Cohesin Complex Component | 1.88776 |
| LRP4 | LDL Receptor Related Protein 4 | 1.799734 |
| LMX1B | LIM Homeobox Transcription Factor 1 Beta | -2.5666115 |
| PDE8B | Phosphodiesterase 8B | -1.7474595 |
| F12 | Coagulation Factor XII | 2.2378125 |
| GIPC1 | GIPC PDZ Domain Containing Family Member 1 | 2.18499375 |
| FZD1 | Frizzled Class Receptor 1 | 2.69894975 |
| IGFBP4 | Insulin Like Growth Factor Binding Protein 4 | 1.683365 |
| PDSS2 | Decaprenyl Diphosphate Synthase Subunit 2 | 1.65552875 |
| PMM2 | Phosphomannomutase 2 | 1.8335025 |
| CCL3 | C-C Motif Chemokine Ligand 3 | 2.45869625 |
| ANXA4 | Annexin A4 | 1.73472875 |
| IRAK4 | Interleukin 1 Receptor Associated Kinase 4 | 1.70402375 |
| DHODH | Dihydroorotate Dehydrogenase (Quinone) | 1.92132075 |
| GAB1 | GRB2 Associated Binding Protein 1 | 1.54629825 |
| TGFBI | Transforming Growth Factor Beta Induced | 7.61696875 |
| PGM3 | Phosphoglucomutase 3 | 2.405959 |
| UBE2D2 | Ubiquitin Conjugating Enzyme E2 D2 | 2.664289 |
| KCNN4 | Potassium Calcium-Activated Channel Subfamily N Member 4 | 1.6491255 |
| DLEU1 | Deleted In Lymphocytic Leukemia 1 | 1.508272 |
| CP | Ceruloplasmin | 1.7426435 |
| AMD1 | Adenosylmethionine Decarboxylase 1 | 1.824881 |
| PRKCA | Protein Kinase C Alpha | 2.71772475 |
| GHSR | Growth Hormone Secretagogue Receptor | -4.49085275 |
| MAL2 | Mal, T Cell Differentiation Protein 2 | -1.657344 |
| PDGFC | Platelet Derived Growth Factor C | 2.50813575 |
| RPS6KA3 | Ribosomal Protein S6 Kinase A3 | 1.51788625 |
